# Increased Release of Apolipoprotein E in Extracellular Vesicles Following Amyloid-β Protofibril Exposure of Neuroglial Co-Cultures

**DOI:** 10.3233/JAD-170278

**Published:** 2017-08-29

**Authors:** Elisabeth Nikitidou, Payam Emami Khoonsari, Ganna Shevchenko, Martin Ingelsson, Kim Kultima, Anna Erlandsson

**Affiliations:** a Department of Public Health and Caring Sciences, Molecular Geriatrics, Rudbeck Laboratory, Uppsala University, Uppsala, Sweden; b Department of Medical Sciences, Clinical Chemistry, Uppsala University Academic Hospital, Uppsala, Sweden; c Department of Chemistry-BMC, AnalyticalChemistry, Uppsala University, Uppsala, Sweden

**Keywords:** Alzheimer’s disease, amyloid-beta peptide, apolipoprotein E, astrocytes, exosomes, extracellular vesicles, mass spectrometry, neurons, shedding microvesicles

## Abstract

Extracellular vesicles (EVs), including exosomes and larger microvesicles, have been implicated to play a role in several conditions, including Alzheimer’s disease (AD). Since the EV content mirrors the intracellular environment, it could contribute with important information about ongoing pathological processes and may be a useful source for biomarkers, reflecting the disease progression. The aim of the present study was to analyze the protein content of EVs specifically released from a mixed co-culture of primary astrocytes, neurons, and oligodendrocytes treated with synthetic amyloid-β (Aβ_42_) protofibrils. The EV isolation was performed by ultracentrifugation and validated by transmission electron microscopy. Mass spectrometry analysis of the EV content revealed a total of 807 unique proteins, of which five displayed altered levels in Aβ_42_ protofibril exposed cultures. The most prominent protein was apolipoprotein E (apoE), and by western blot analysis we could confirm a threefold increase of apoE in EVs from Aβ_42_ protofibril exposed cells, compared to unexposed cells. Moreover, immunoprecipitation studies demonstrated that apoE was primarily situated inside the EVs, whereas immunocytochemistry indicated that the EVs most likely derived from the astrocytes and the neurons in the culture. The identified Aβ-induced sorting of apoE into EVs from cultured neuroglial cells suggests a possible role for intercellular transfer of apoE in AD pathology and encourage future studies to fully elucidate the clinical relevance of this event.

## INTRODUCTION

Alzheimer’s disease (AD) is a progressive neurodegenerative disorder and the leading cause of dementia worldwide. The key neuropathological hallmarks in AD are extracellular senile plaques, mainly consisting of aggregated amyloid-β (Aβ), and intracellular neurofibrillary tangles, composed of hyperphosphorylated tau (p-tau). The accumulation of Aβ in the AD brain is assumed to be caused by an imbalance between Aβ production and Aβ clearance. Since the majority of patients with sporadic, late-onset AD do not have an increased Aβ production, insufficient lysosomal degradation has been suggested to be the most common disease mechanism [1–3]. Due to their hydrophobic nature, Aβ monomers aggregate and form soluble Aβ species, which eventually deposit as senile plaques. Data from several research groups suggest that the widespread neuronal dysfunction in the AD brain is caused by soluble oligomers and protofibrils, rather than the insoluble fibrils [4, 5]. Prefibrillar forms of Aβ species have been shown to be the predominant species of soluble Aβ aggregates in both transgenic mice and human AD brains [6, 7]. Moreover, elevated levels of soluble Aβ aggregates in cerebrospinal fluid (CSF), correlating with cognitive impairment, have been demonstrated in AD patients compared to controls [8–11].

Apolipoprotein E (apoE) is the major transporter of cholesterol and phospholipids between cells [12]. In humans, there are three apoE isoforms and carrying apoE *ɛ*4 is one of the strongest genetic risk factors in developing late-onset AD [13]. Epitope mapping has demonstrated that residues on the N-terminal part of apoE *ɛ*4 can interact with the Aβ peptide [14]. Moreover, apoE *ɛ*4 has been shown to be less effective in clearing Aβ peptides, compared to apoE *ɛ*2 (which is considered to be neuroprotective) and apoE *ɛ*3 [15].

We have previously developed a cell culture model, suitable for studies of Aβ-induced pathology, based on Aβ_42_ protofibril exposure of primary astrocytes, neurons, and oligodendrocytes [16]. Using this model, we recently demonstrated that the astrocytes engulf and accumulate large amounts of Aβ_42_ protofibrils. The intracellular storage of Aβ results in severe astrocytic endosomal/lysosomal defects and secretion of extracellular vesicles (EVs) with neurotoxic content [16].

Here, we have used the same co-culture system with the aim to analyze the protein content of EVs, specifically released from cells exposed to Aβ_42_ protofibrils. The EVs include vesicles of various sizes that are formed and secreted through slightly different mechanisms. Larger microvesicles (MVs; 100–1000 nm) originate and bud directly from the plasma membrane of cells, whereas exosomes (40–100 nm) are generated from endosomal multivesicular bodies (MVBs) that fuse with the plasma membrane [17–20]. Apoptotic bodies (800–5000 nm) are another sort of EVs that are specifically released by cells undergoing apoptosis [21]. EVs can be isolated from cell culture medium and various body fluids, including plasma, CSF and urine [22–24]. The delivery of EVs from one cell to another is known to constitute an important form of intercellular communication, distributing proteins, lipids, nucleic acids, and sugar molecules [25]. However, the role of EVs in AD progression and the material that EVs carry in response to Aβ_42_ pathology is poorly understood. The presence of Aβ and amyloid-β protein precursor (AβPP) in EVs has been identified for several cell lines [26, 27] and has been confirmed in CSF from cynomolgus monkeys and AβPP transgenic mice, respectively [28]. Since the EV content mirrors the intracellular environment, it is believed to change during disease progression and could therefore be a rich source for dynamic biomarkers [29].

In order to develop effective therapeutic interventions for AD patients, a better knowledge of the basic cellular mechanisms underlying the disease progression is crucial. In the current study, we show that the release of EVs did not vary in response to Aβ_42_ protofibril exposure, although the protein content in the EVs changed markedly. Using mass spectrometry (MS) and western blot analysis, we identified a threefold increase of apoE in EVs from Aβ_42_ protofibril exposed cells, compared to unexposed cells. The apoE was primarily situated inside the EVs and were most likely secreted by the astrocytes and neurons in the culture.

## MATERIALS AND METHODS

### Synthetic Aβ_42_ protofibrils

Aβ_42_ protofibrils were produced from synthetic unlabeled Aβ_42_ peptides (American Peptide Company Inc.) according to a well-established protocol [6, 30–32]. These Aβ_42_ protofibrils have previously been carefully characterized in our laboratory, using different methods, including HPLC-SEC, electron microscopy, Thioflavin T staining, and ELISA [6, 16, 30–32]. In brief, the Aβ_42_ peptides were dissolved in 10 mM NaOH and mixed with 10 *x* peptide-PBS to a concentration of 443*μ*M (2 mg/ml). The mixture was incubated for 30 min at 37C, followed by centrifugation for 5 min at 17,900 *x g* at 4C to remove any insoluble aggregates. The supernatant was diluted 1:4 in sterile PBS to a final concentration of 0.5 mg/ml. Analysis of the synthetic Aβ_42_ peptides was routinely performed, using the protofibril specific ELISA, mAb158 ELISA, to confirm the expected concentration.

### Animals

All experimental animal procedures were approved by the Uppsala County Animal Ethics Board (ethical permit number: C75/13, valid 2013-06-28 to 2018-06-28) in Uppsala, Sweden and followed the current National and European guidelines for animal research. Female C57/BL6 mice were housed in a 12 h dark-light cycle and had *ad libitum* access to food and water.

### Neural cell cultures

Cerebral cortices were dissected from C57/BL6 mice embryos (E14) in Hank’s buffered salt solution (HBSS) supplemented with 50 U/ml Penicillin, 50 mg/ml Streptomycin, and 8 mM Hepes buffer (all from ThermoFisher Scientific). The cortices were centrifuged in fresh HBSS for 3 min at 150 *x g* and then resuspended and dissociated into a homogenous solution. Any remaining blood vessels were allowed to sediment for 10 min. The supernatant was transferred to a new tube and centrifuged for 5 min at 150 *x g*. The cell pellet was carefully resuspended in DMEM/F12-GlutaMAX cell culture medium supplemented with 50 U/ml Penicillin, 50 mg/ml Streptomycin, 8 mM Hepes buffer, 1 *x* B27 supplement, 10 ng/ml bFGF2 (all from ThermoFisher Scientific), and 20 mg/ml EGF (VWR). Cells were expanded as neurospheres in flasks (ThermoFisher Scientific) and were passaged every second or third day by dissociation in HBSS and resuspension in cell culture medium with bFGF2 and EGF. When the number and size of neurospheres was adequate, the cells were plated as a monolayer at a concentration of 30 000 cells/cm^2^ on Poly-L-Ornithine (Sigma-Aldrich) and Laminin (Invitrogen) coated coverslips (*In Vitro* Diagnostics) in 6-well plates (Falcon) or coated cell culture dishes (Corning). Cells were cultured in medium containing bFGF2 and EGF for 24 h. The medium was then replaced with mitogen-free medium to initiate differentiation to a mixed co-culture of astrocytes, neurons and oligodendrocytes, but not microglia (since they originate from the hematopoietic stem cell line). During the 7-day differentiation period, medium was changed every second to third day. Only neurospheres from passage 2–4 were used for the experiments. Independent cell cultures were derived from embryos of different pregnant mice.

### Aβ_42_ protofibril exposure

Differentiated co-cultures of astrocytes, neurons, and oligodendrocytes were first washed in phenol-free media and then exposed to 0.1*μ*M Aβ_42_ protofibrils in phenol-free DMEM/F-12 media containing Penicillin, Streptomycin, and Hepes buffer, but no B27 supplement (phenol-free medium without B27 supplement was used to prevent any interference with the MS analysis). Parallel control cultures received the same media without Aβ_42_ protofibrils. The cell culture media was collected two days after constant exposure (day 0–2) and replaced with fresh media (with or without 0.1*μ*M Aβ_42_ protofibrils) for an additional three days of culture prior to the second media collection (day 2–5). Ultracentrifugation was performed within 1 h following media collection.

### Isolation of EVs

**Fig.1:**
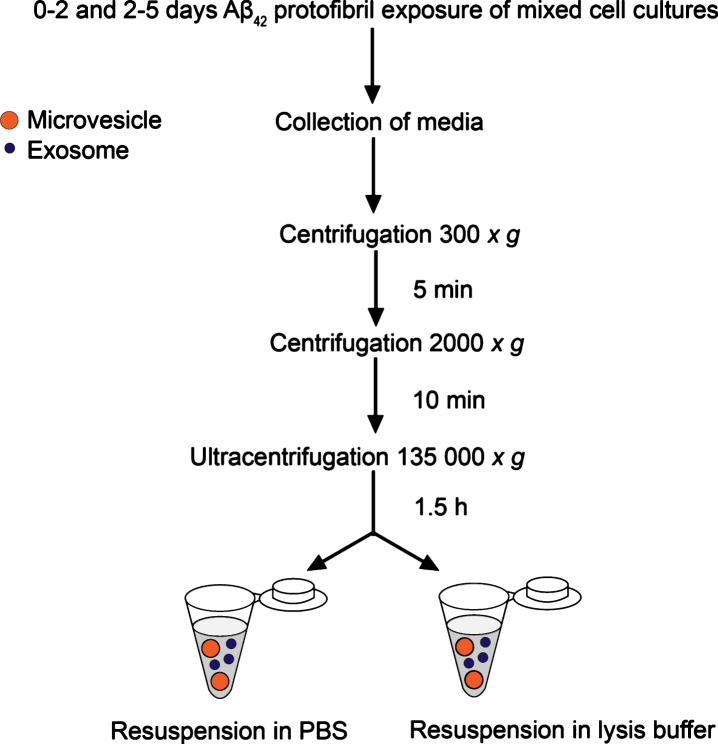
Study set-up for ultracentrifugation procedure. Cells were incubated with medium containing Aβ_42_ protofibrils or control medium for 5 days. Medium was collected after two days (day 0–2) and replaced with new medium. The cell cultures were incubated for additional three days, prior to the second medium collection (day 2–5). The medium was centrifuged in two steps at lower speed followed by ultracentrifugation for 1.5 h at 135,000 *x g*. The pellets were resuspended in PBS for transmission electron microscopy, or in lysis buffer with protease inhibitors for MS and western blot analysis.

Figure 1 shows a schematic outline of the experimental set-up. A total volume of 32 ml medium for each cell culture batch was collected and centrifuged for 5 min at 300 *x g* to remove free-floating cells, followed by centrifugation for 10 min at 2000 *x g* to remove any remaining cell remnants. The supernatants were transferred to Ultra-Clear centrifuge tubes (Beckman Coulter) and ultracentrifuged using a SW28 rotor in a Beckman LE-80 ultracentrifuge for 1.5 h at 135,000 *x g* at 4C, to obtain a pellet of EVs, including larger MVs and exosomes (Fig. 1). The pellets were resuspended in either 330*μ*l PBS for transmission electron microscopy or in ice-cold lysis buffer (20 mM Tris pH 7.5, 0.5% Triton X-100, 0.5% deoxycholic acid, 150 mM NaCl, 10 mM EDTA, 30 mM NaPyroP and protease inhibitor cocktail (ThermoScientific)) for MS and western blot analysis. The samples used for transmission electron microscopy were kept at 4C for maximum three days prior to sample preparation and EV lysates were stored at –70C until analysis. In order to discriminate between exosomes and larger MVs, we performed experiments in which the samples (following the two initial centrifugation steps) were first ultracentrifuged for 30 min at 10,000 *x g* at 4C (to pellet MVs). The supernatant was then ultracentrifuged a second time for 1.5 h at 135,000 *x g* at 4C (to pellet exosomes). The MV and exosome pellets were resuspended in 330*μ*l lysis buffer.

### Immunocytochemistry

Cell cultures were fixed in 4% paraformaldehyde for 15 min at room temperature (RT), permeabilized and blocked in 0.1% Triton X-100 with 5% normal goat serum (NGS) in phosphate-buffered saline (PBS) for 30 min at RT. Primary antibodies were diluted in 0.1% Triton X-100 with 0.5% NGS in PBS and added to the cells for 1–4 h at RT. Primary antibodies used in the study were: rabbit anti-Glial Fibrillary Acidic Protein (GFAP, 1:400, DakoCytomation), mouse anti-GFAP (1:400, Sigma-Aldrich), mouse anti-2’,3’-Cyclic-nucleotide 3’-phosphodiesterase (CNPase, 1:500, Sigma-Aldrich), rabbit anti-CNPase (1:100, Sigma-Aldrich), mouse anti-βIII-tubulin (1:200, Covance), polyclonal rabbit anti-Aβ_42_ (1:200, Invitrogen), mouse anti-apoE (1:1000, Abcam), rabbit anti-CD9 (1:200, Sigma-Aldrich), and rabbit anti-GM-160 (1:200, Abcam). Coverslips were washed three times in PBS prior to incubation of secondary antibodies for 45 min-1 h at 37C. Secondary antibodies used were AlexaFluor 488 and 555 against mouse or rabbit (1:200, Molecular Probes). After additional washing steps, the coverslips were mounted on microscope glass slides using Vectashield hard-set mounting medium with DAPI (DAKO). TUNEL assays (Roche) were performed according to the manufacturer’s instructions to quantify the number of apoptotic cells. A Zeiss Observer Z1 microscope (Zeiss) was used for analysis and images were visualized with Zen 2012 software.

### Cell counting

The percentage of astrocytes, neurons, and oligodendrocytes in the culture was performed by manually counting the number of GFAP, βIII-tubulin and CNPase positive cells from 30 systematically captured images (10 images/cover slip from three independent cultures). Three independent cell culture experiments were performed with cells derived from embryos of different pregnant mice. The data are presented as the number of living neurons, astrocytes, and oligodendrocytes relative to the number of each cell type in unexposed, control cultures. The total number of living cells was quantified by counting the number of cells, with non-condensed nuclei from 90 systematically captured images (10 images/cover slip from 9 independent cultures).

To investigate whether lack of B27 supplement in the media had a negative impact on cell survival, TUNEL assays were performed on fixed Aβ_42_ protofibril exposed and unexposed cells from day 0, 2, and 5 following treatment. Ten systematically taken images/coverslip were analyzed by manually quantifying the number of TUNEL positive cells and the number of living DAPI nuclei (non-condensed nuclei with visible chromatin and not stained for TUNEL). Two independent cell culture experiments were performed with cells derived from embryos of different pregnant mice. The data are presented as the relative number of TUNEL positive cells and relative number of live cells, compared to the control cultures (that received B27).

### Transmission electron microscopy

#### Cells

Cells were briefly washed in PBS prior to fixation in 2.5% glutaraldehyde (1 h or overnight at 4C). The dishes containing the cells were rinsed with 0.15 M sodium cacodylate buffer (pH 7.2–7.4) for 10 min, and incubated in newly prepared 1% osmium tetraoxide in 0.1 M sodium cacodylate for 1 h at RT. Sodium cacodylate was rinsed away to dehydrate the dishes with 70% ethanol for 30 min, 95% ethanol for 30 min and >99% ethanol for 1 h. Following dehydration, a thin newly made plastic layer (Agar 100 resin kit, Agar Scientific Ltd) was added to the dishes and incubated for 1 h to permit evaporation of the alcohol. The plastic was poured off and a new plastic layer was added onto the dishes for incubation O/N in a desiccator. Next, the plastic was heated to enable its removal and a new thicker plastic layer was added and dishes were incubated for 1 h in a desiccator. Cells were covered with a ∼3 mm plastic and polymerized in oven at 60C for 48 h. By using a Leica ultracut UTC ultrotome (Rowaco AB), embedded cells were sectioned and studied in a Tecnai G2 transmission electron microscope (FEI Company) with an ORIUS SC200 CCD camera and Gatan Digital Micrograph software (both from Gatan Inc.).

#### EVs

EVs were isolated by ultracentrifugation and resuspended in PBS as described above. Samples were added onto a formvar-coated 200-mesh grid (Oxford 11 Instruments) and incubated for 45 min. The grid was dried by transversely moving it against a filter paper. Immediately, 1% uranyl acetate was added onto the grid followed by 10 s incubation. The grid was dried for at least 15 min before analysis in a Tecnai G2 transmission electron microscope (FEI Company) with an ORIUS SC200 CCD camera and Gatan Digital Micrograph software (both from Gatan Inc.).

### Chemicals and reagents for Mass spectrometry analysis

Acetonitrile (ACN), methanol (MeOH), acetic acid (HAc), formic acid (FA), and ammonium bicarbonate (NH_4_HCO_3_) were obtained from Merck. Protease inhibitor cocktail and trifluoroacetic acid (TFA) were purchased from Sigma-Aldrich. For tryptic digestion, iodoacetamide (IAA), urea, and dithiothreitol (DTT) were obtained from Sigma-Aldrich and trypsin/Lys-C mixture (mass spectrometry grade; Promega) were used. Ultrapure water was prepared by Milli-Q water purification system (Millipore).

### Protein quantification for Mass spectrometry analysis

The total protein content of the extracted samples was determined using the DC Protein Assay Kit (BioRad Laboratories), which is based on the modified Lowry method with bovine serum albumin (BSA) as standard [33]. The DC assay was carried out according to the manufacturer’s instructions using a 96-well microtiter plate reader model 680 (BioRad Laboratories).

### On-filter desalting and digestion of proteins in EVs resuspended in lysis buffer

Aliquots of 140*μ*l of EVs dissolved in lysis buffer were processed with a previously described on-filter digestion protocol [34] using 3 kDa centrifugal filters (Millipore). The samples were reduced and alkylated in 1.5 ml protein low bind Eppendorf tubes prior the on-filter desalting and digestion. Centrifugation was carried out using a centrifugal force of 14,000 *x g* throughout the protocol. A volume of 10*μ*l of 45 mM aqueous DTT was added to all samples and the mixtures were incubated for 15 min at 50C to reduce the disulfide bridges. The samples were cooled down to RT and 10*μ*l of 100 mM aqueous IAA was added and the mixtures were incubated for an additional 15 min at RT in darkness to carabamidomethylate the cysteines. The samples were transferred to spin filters that had been pre-washed with 250*μ*l of 50% ACN for 15 min and then 500*μ*l of water for 20 min. The samples were then centrifuged for 10 min to remove the added salts, detergents, and other interfering substances. An additional volume of 100*μ*l of 2% ACN in 50 mM NH_4_HCO_3_ was added and the filters were spun for 10 min followed by 100*μ*l of 50:50 ACN: 50 mM NH_4_HCO_3_ and 100*μ*l of 50 mM NH_4_HCO_3_, and centrifugation for another 10 min. Finally, a volume of 100*μ*l of 50 mM NH_4_HCO_3_ was added together with trypsin/Lys-C mixture to yield a final trypsin/protein concentration of 5% (w/w). The tryptic digestion was performed at 37C O/N in darkness. The samples were then centrifuged for 20 min to collect the tryptic peptides in the filtrate while retaining undigested proteins and trypsin. An additional volume of 100*μ*l of 50% ACN, 1% HAc was added and the filters were spun for 10 min and pooled with the first tryptic peptide filtrate. The collected filtrates were vacuum centrifuged to dryness using a Speedvac system ISS110 (ThermoFisher Scientific). Prior to nanoLC-MS/MS analysis, samples were redissolved in 0.1% TFA to yield an approximate tryptic peptide concentration of 0.3*μ*g/*μ*l.

### NanoLC-MS/MS for protein identification

The protein identification experiments were performed using a 7 T hybrid LTQ FT mass spec-trometer (ThermoFisher Scientific) fitted with a nano-electrospray ionization (ESI) ion source. On-line nanoLC separations were performed using an Agilent 1100 nanoflow system (Agilent Technologies). The peptide separations were performed on in-house packed 15-cm fused silica emitters (75-*μ*m inner diameter, 375-*μ*m outer diameter). The emitters were packed with a methanol slurry of reversed-phase, fully end-capped Reprosil-Pur C_18_-AQ 3*μ*m resin (Dr. Maisch GmbH) using a pressurized packing device operated at 50–60 bars. The separations were performed at a flow of 200 nl/min with mobile phases A (water with 0.5% acetic acid) and B (89.5% acetonitrile, 10% water, and 0.5% acetic acid). A 100-min gradient from 2% B to 50% B followed by a washing step with 98% B for 5 min was used. Mass spectrometric analyses were performed using unattended data-dependent acquisition mode, in which the mass spectrometer automatically switches between acquiring a high resolution survey mass spectrum in the FTMS (resolving power 50,000 FWHM) and consecutive low-resolution, collision-induced dissociation fragmentation of up to five of the most abundant ions in the ion trap.

### Data analysis of Mass spectrometry

The raw data from MS was converted to open source format (mzML) by “msconvert” from ProteoWizard [35] and processed using an automated label free pipeline in OpenMS platform (with the exception that FeatureFinderMultiplex was used instead of FeatureFinderCentroided) using the following parameters (unmentioned parameters were set to default): FeatureFinderMultiplex: Mz tolerance: 10 ppm, rt_min: 2, rt_typical: 35, intensity_cutoff: 2000, low charge: 2, high charge: 4; IDMapper: RT tolerance: 5 seconds, Mz tolerance: 20 ppm; MapAlignerIdentification: RT: 120 seconds; FeatureLinkerUnlabeledQT: 10 ppm, RT: 60 s. Identification was performed using MSGFPlus on UniProt/Swiss-Pro human database (release 2014_03) combined with a decoy database (reversed sequences) using the following parameters; Enzyme: Trypsin, missed cleavages: 2 precursor mass tolerance: 10 ppm, minimum charge: 2, maximum charge: 4, maximum modifications: 3, Carbamidomethyl (C), variable modifications: Oxidation (M) and Deamidated (N and Q). The peptide matches with q-value lower than 0.05 were used in Fido to score proteins based on peptide-spectrum matches. The proteins with q-value lower than 0.05 were selected for subsequent analysis. The “IDConflictResolver” was used to filter the identification such that each feature is associated to only one identification hit. Finally, peptide abundances were aggregated to protein abundance using “ProteinQuantifier”, in which the intensity of the peptides (with peptide and protein q-value lower than 0.05) were summed and the result was imported to R [36] and log_2_ transformed for further processing.

### Statistical analysis from peptide quantification of Mass spectrometry

To perform statistical testing on the proteins, within each exposure time (day 0–2 and day 2–5), every protein abundance in the exposed sample was subtracted by its corresponding abundance in the control state (the culture without Aβ_42_ protofibril exposure). For normalization, the protein abundances (paired samples) were subtracted by the sample mean (centering) and divided by the standard deviation of the sample (scaling). Multiple linear models were fitted through all the peaks using “lmFit” (robust method was used in lmFit) and the “duplicateCorrelation” function [42] was used to estimate the correlation between the technical replicates. Finally, moderated t-statistics and F.*p*-value were calculated using the “ebayes” function [43]. The F.*p*-values were converted to q-value (referred to F.q-value) and the proteins (characterized with more than ten peptides) with F.q-value lower than 0.05 and at least two observations (out of three biological replicates) were selected for further analysis.

### Cell lysis

Cell culture medium was carefully removed from the dishes, washed one time in PBS, and put on ice. Lysis buffer with protease inhibitors was added to each dish, scraped using a cell lifter (Corning) and transferred to protein LoBind tubes (Eppendorf). Samples were kept on ice for 30 min prior to centrifugation at 10,000 *x g* for 10 min at 4C. The supernatants were transferred to new tubes and stored in –70C until analysis.

### Western blot analysis

The protein concentration of lysed cells and EVs was measured with Pierce BCA protein kit (ThermoScientific), according to the manufacturer’s instructions. In total, 18*μ*g protein of cell lysates and 5*μ*g protein of lysed EVs was mixed with Bolt LDS Sample buffer and Sample Reducing agent (both from ThermoFisher Scientific) and incubated for 5 min at 95C to denature the proteins. Samples were loaded on Bolt 4–12% Bis-Tris plus gels and run in Bolt MES SDS running buffer (both from ThermoFisher Scientific) for 22 min at 200 V. Chameleon duo protein ladder or Chameleon Kit 700 and 800 pre-stained protein ladder (Li-Cor) were used. The proteins were transferred to PVDF membranes (1 h, at 20 V) in Bolt transfer buffer (both from ThermoFisher Scientific), containing 10% methanol, 0.1% Bolt antioxidant (ThermoFisher Scientific), and 0.01% sodium dodecyl sulfate (SDS). The membranes were blocked in 5% BSA (Sigma-Aldrich) in 0.1% TBS-T for 1 h on shaking at RT and then incubated with primary antibody in 0.5% BSA in 0.1% TBS-T O/N at 4C. Primary antibodies used in the study were: rabbit anti-apoE (1:1000, Novus Biologicals), mouse anti-flotillin-1 (1:500, BD Biosciences), rabbit anti-CD9 (1:500, Sigma-Aldrich), rabbit anti-Tumour susceptibility gene 101 (TSG101, 1:500, Abcam). After extensive washes in TBS-T, the membranes were incubated with horseradish peroxidase-conjugated (HRP) secondary goat anti-rabbit or goat anti-mouse antibody (1:20 000, Pierce) in 0.5% BSA in 0.1% TBS-T for 1 h on shaking at RT. Immunoreactive bands were visualized with enhanced chemiluminescence (ECL, GE Healthcare) using a ChemiDoc XRS with Image Lab Software for intensity measurements of bands (Bio-Rad Laboratories). A total of six independent western blot experiments with the apoE and flotillin-1 antibodies were performed on the lysed EVs and cell lysates, respectively, for quantitative analysis. A control experiment was performed to exclude that the vehicle (NaOH+PBS), used during the Aβ_42_ protofibril production, had any impact on the results. Western blot analysis confirmed that there was no difference in apoE expression in EVs from control cultures and the cultures treated with vehicle (data not shown).

### Immunoprecipitation

Following ultracentrifugation of 32 ml media from Aβ_42_ protofibril exposed cultures, the pellets were resuspended in 400*μ*l PBS to obtain intact EVs, or in 400*μ*l lysis buffer with protease inhibitors to lyse all EVs and expose their content. Half of each EV sample (200*μ*l) was mixed with 10*μ*g goat anti-apoE antibody (Santa Cruz Biotechnology) in antibody binding buffer (ThermoFisher Scientific) and incubated on slow shaking O/N at 4C. Then, 3 mg Dynabeads protein G (ThermoFisher Scientific) was added to each sample and incubated on slow shaking for 2 h at RT. Immunoprecipitation was performed using a magnetic particle concentrator (Dynal Biotech) and the samples were then carefully washed three times in washing buffer (ThermoFisher Scientific). After, 20*μ*l elution buffer (ThermoFisher Scientific) and 10*μ*l premixed Bolt LDS Sample buffer (diluted in lysis buffer with protease inhibitors and Sample Reducing agent, ThermoFisher Scientific) was added to each sample. The samples were gently mixed and heated for 5 min in 95C. The supernatants (30*μ*l) were loaded on a Bolt 4–12% Bis-Tris plus gel for western blot analysis (described above). Primary antibody was rabbit anti-apoE (1:1000, Novus Biologicals) and secondary antibody was HRP-conjugated Natural Protein G (1:10 000, Abcam). In total, three independent western blot analyses were performed with cells derived from embryos of different pregnant mice.

### Statistical analysis of cell counting and western blot data

All the results are presented in scatter plots or box plots with mean±standard deviation. Since the data was found not to meet the assumption of normal distribution using the Shapiro-Wilk’s W-test, non-parametric tests were selected for analysis. For the cell counting data, statistical analysis was performed using Mann-Whitney *U*-test. Statistical analysis of western blot data was performed using Mann-Whitney *U*-test for comparisons of unpaired data and Wilcoxon matched-pairs signed rank test for comparisons of paired data. Differences between the groups were considered to be significant at *p < 0.05, **p < 0.01 and ***p < 0.001.

## RESULTS

### Aβ_42_ protofibrils are ingested and accumulated by astrocytes

**Fig.2:**
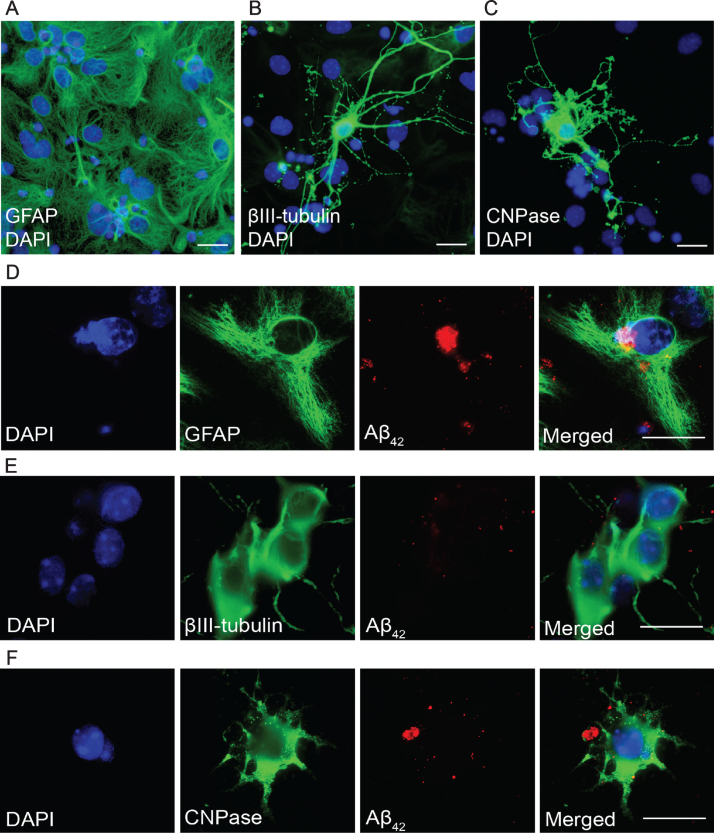
Aβ_42_ protofibrils are accumulated in astrocytes. Immunocytochemistry demonstrating the three cell types in the unexposed co-culture system; astrocytes (A), neurons (B), and oligodendrocytes (C). Astrocytes exposed to Aβ_42_ protofibrils for 2 days contained large deposits of Aβ_42_ (D). Neurons lacked detectable levels of intracellular Aβ_42_ (E), but the few oligodendrocytes in the culture showed some Aβ_42_ accumulation (F). DAPI (blue), GFAP, βIII-tubulin, CNPase (all green), and Aβ_42_ (red). Scale bars: A-F = 20*μ*m.

In the present study, we have used an *in vitro* model of AD, based on Aβ treatment of mixed cortical mouse cells. Astrocytes are the most abundant cell type (70%) in this cell system, followed by neurons (25%) and oligodendrocytes (5%) [16]. The co-cultures were exposed to Aβ_42_ protofibrils, or left untreated, for 2 days prior to fixation and immunostainings with antibodies against Aβ_42_ in combination with specific markers for astrocytes (GFAP, Fig. 2A), neurons (βIII-tubulin, Fig. 2B), and oligodendrocytes (CNPase, Fig. 2C). Although the media used in this study differed from our previous investigations (lacking phenol-red and B27 supplement), the three cell types in the culture behaved similarly as in our previous studies [16]. Accordingly, addition of Aβ_42_ protofibrils to the cell cultures resulted in large Aβ_42_ deposits in the astrocytes (Fig. 2D). The Aβ_42_ did not accumulate in neurons (Fig. 2E), but to some extent in oligodendrocytes (Fig. 2F).

### Aβ_42_ protofibril exposure has no direct effect on neuronal death

**Fig.3:**
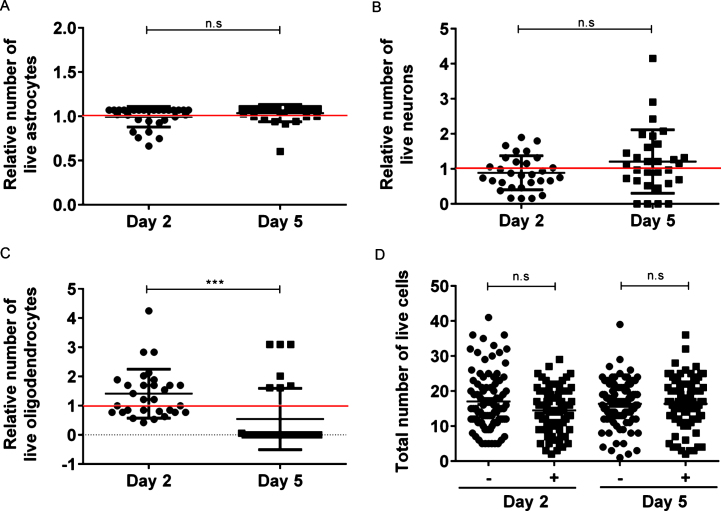
Aβ_42_ protofibril exposure does not directly induce neuronal cell death. The number of living cells was manually quantified for each of the three cell types after 2 and 5 days of Aβ_42_ protofibril exposure and plotted as the relative value, normalized to the living cells of respective cell type in unexposed cultures. The red lines represent 1, i.e., unchanged value, as compared to control. The relative number of viable astrocytes did not change significantly between the two time points (*p* = 0.06) (A). Relative number of viable astrocytes day 2: 0.995±0.116, day 5: 1.036±0.097. Furthermore, Aβ_42_ protofibrils did not affect the relative viability of neurons at day 2 (*p* = 0.38) or at day 5 (*p* = 0.55), and there was no change between the two time points (*p* = 0.11) (B). Relative number of viable neurons day 2: 0.887±0.485, day 5: 1.208±0.906. The relative number of viable oligodendrocytes significantly decreased from day 2 to day 5 (***p < 0.001) (C), most likely due to higher Aβ sensitivity when cultured without B27 supplement. Relative number of viable oligodendrocytes day 2: 1.411±0.836, day 5: 0.543±1.052. All experiments were performed in triplicates with independent cell cultures and 10 images per experiment were analyzed with Wilcoxon matched-pairs signed rank test. Moreover, Aβ_42_ protofibril treatment did not affect the total viability of cells in the co-culture, neither at day 2 (*p* = 0.11) nor at day 5 (*p* = 0.97) (D). Each dot in the graph represents the total number of living cells from one image. The experiments were performed with 9 independent cell cultures and 10 images per experiment were analyzed with Mann-Whitney *U*-test.

To further investigate how exposure of Aβ_42_ protofibrils affects the viability of cells, we manually quantified the number of living astrocytes, neurons and oligodendrocytes in the culture 2 and 5 days after exposure to Aβ_42_ protofibrils, as well as in parallel untreated cultures. We found that there was no change in the relative number of astrocytes in the cultures between day 2 and day 5 (*p* = 0.06, Fig. 3A), as compared to untreated control cultures. Neither did the relative number of living neurons change over the 5-day period (*p* = 0.11, Fig. 3B). Moreover, there was no difference in neuronal death between exposed and unexposed cultures, demonstrating that Aβ_42_ protofibrils did not affect the viability of neurons in the co-culture system over the 5-day period (*p* = 0.55, Fig. 3B). Unlike our previous findings, where we observed no decrease in survival of oligodendrocytes following Aβ_42_ protofibril exposure, the viability of the oligodendrocytes in these cultures were significantly decreased (*p* < 0.001, Fig. 3C), most likely due to higher Aβ sensitivity of oligodendrocytes, when cultured without B27 supplement in the media. However, quantification of the total number of living cells (all three cell types included) revealed no statistically significant differences at the two time points (day 2: *p* = 0.11 and day 5: *p* = 0.97, Fig. 3D). Moreover, analysis of the whole culture using TUNEL assay, demonstrated that there was no significant increase in apoptosis as a result of the removal of B27 supplement from the cell culture media (Supplementary Figure 1A-E).

### Secreted EVs were successfully isolated from neuroglial co-cultures

**Fig.4:**
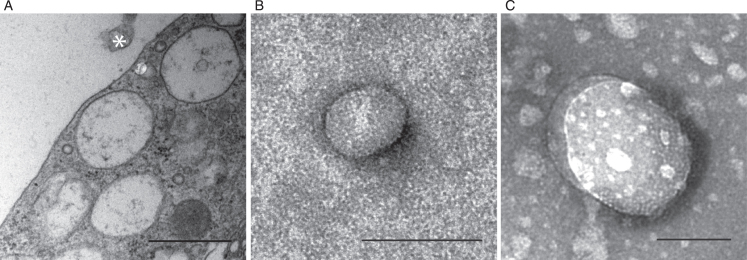
Secreted EVs can be successfully isolated from neuroglial co-cultures. Transmission electron microscopy confirming the presence of an EV (asterisk) in close proximity to multivesicular astrocytes (A). The EV preparation included smaller vesicles, such as exosomes (B) and larger MVs (C). The size of the exosomes ranged from 50–75 nm and the larger MVs were up to 400 nm in diameter. Scale bars: *A* = 1*μ*m, B-C = 100 nm.

Aβ_42_ protofibrils were added to the cells and constantly incubated for 2 (day 0–2) and 5 (day 2–5) days. Media was collected, centrifuged in two steps followed by ultracentrifugation. By transmission electron microscopy, we found EVs that were in close proximity to multivesicular astrocytes (Fig. 4A). However, this does not exclude that the EVs can be secreted by the other cells in the co-culture. Both smaller vesicles, such as exosomes (Fig. 4B), and larger MVs (Fig. 4C) were present in the pellet fraction of the medium after ultracentrifugation. The size of the exosomes ranged from 50–75 nm and the larger MVs were up to 400 nm in diameter. Successful isolation of EVs enabled us to further investigate and compare the protein content in EVs released from unexposed and Aβ_42_ protofibril exposed cells.

### Proteins with altered levels in EVs following Aβ_42_ protofibril exposure

**Fig.5:**
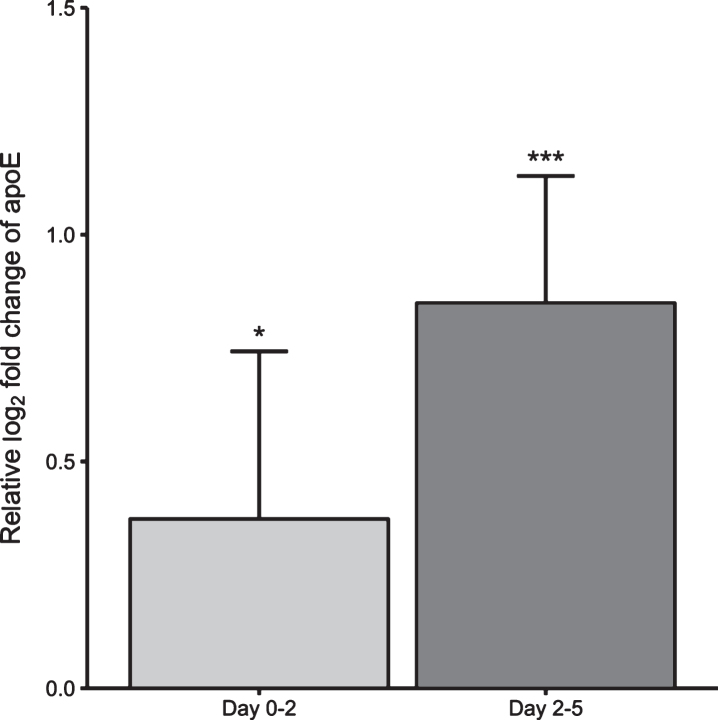
Proteins with altered levels in EVs following Aβ_42_ protofibril exposure. Mass spectrometry analysis on the protein content in EV lysates revealed 807 unique proteins in total (q-value < 0.05). After further statistical testing and strict quality control, five proteins were identified to be significantly differentially expressed in EVs from Aβ_42_ protofibril exposed cultures, compared to unexposed cultures for any of the two time points (day 0–2 and day 2–5). The only protein that showed significantly altered levels and at both time points was apoE. Data is presented as relative fold change of apoE in Aβ_42_ protofibril exposed versus unexposed cultures and was performed in three independent cell culture experiments.

Label-free shotgun MS-based proteomics was performed to examine the protein content in EV lysates. A total of 807 proteins were identified with q-value lower than 0.05 (Supplementary Table 1). After performing statistical testing and quality control, five proteins with two or more observations and characterized with more than ten peptides were found to have F.q-value lower than 0.05 (Supplementary Table 2). These proteins were apolipoprotein E (apoE), 2’,3’-cyclic-nucleotide 3’-phosphodiesterase (CNPase), clathrin heavy chain 1, 60S ribosomal protein L4, and cytoplasmic dynein 1 heavy chain 1. All these proteins are well-described and are involved in central cellular processes, but they are not known to be directly linked to each other. The apoE protein is the major transporter of cholesterol and phospholipids between cells [12]. CNPase is a myelin-associated enzyme that is expressed exclusively by oligodendrocytes. In addition to its myelin related function, CNPase is involved in local adenosine production and may have a regulatory function in mitochondrial membrane permeabilization [37]. Clathrin is a cytoplasmic protein that has a central role in endocytosis and intracellular trafficking [38]. The 60S ribosomal protein L4 is one of the units in the complex ribosomal machinery [39]. The dyneins are a group of microtubule-activated ATPases that function as molecular motors. The cytoplasmic dyneins, i.e., cytoplasmic dynein 1, are important for intracellular motility, including retrograde axonal transport, protein sorting, organelle movement, and spindle dynamics [40]. For these five proteins, we further extracted *p-value* and fold changes for individual contrasts for day 0–2 and 2–5 (each reflecting differences in Aβ_42_ protofibril exposed cultures, compared to unexposed cultures in the corresponding time). Interestingly, apoE stood out particularly among all proteins and showed a significantly higher expression level (*p* < 0.05) in EVs derived from Aβ_42_ protofibril exposed cells compared to unexposed cells, both at day 0–2 and day 2–5 (Fig. 5). The other four proteins showed significantly altered levels only in day 0–2 or day 2–5 (not both of them).

### Apolipoprotein E is significantly increased in EVs following Aβ_42_ protofibril exposure

**Fig.6:**
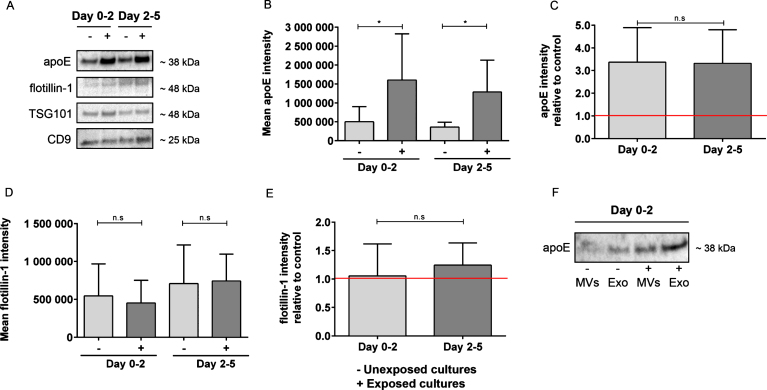
Increased apoE levels in EVs following Aβ_42_ protofibril exposure. Intravesicular expression levels of apoE were measured by western blot analysis. Apolipoprotein E was found to be highly increased in response to Aβ_42_ protofibril exposure at both day 0–2 and day 2–5 (*p = 0.03 for both time points) (A,B). The mean apoE intensity was 5.0E5±4.0E5 at day 0–2 in unexposed samples and 3.6E5±1.3E5 at day 2–5 in unexposed samples (B). The mean apoE intensity was 16.0E5±12.2E5 at day 0–2 in Aβ_42_ protofibril exposed samples and 12.9E5±8.5E5 at day 2–5 in Aβ_42_ protofibril exposed samples (B). The relative abundance of apoE to control remained unchanged over time (*p* = 0.69) (C). The increase in apoE intensity relative to control was 3.4±1.5 and 3.3±1.5 at day 0–2 and day 2–5, respectively. The amount of released EVs was also examined by western blot analysis with antibodies to flotillin-1, TSG101 and CD9. There was no change in flotillin-1 expression between Aβ_42_ protofibril exposed and unexposed cultures (D), neither at day 0–2 (*p* = 0.94), nor at day 2–5 (*p* = 0.59) (D). The mean flotillin-1 intensity was 5.5E5±4.2E5 at day 0–2 in unexposed samples and 7.1E5±5.1E5 at day 2–5 in unexposed samples (D). The mean flotillin-1 intensity was 4.5E5±3.0E5 at day 0–2 in Aβ_42_ protofibril exposed samples and 7.4E5±3.6E5 at day 2–5 in Aβ_42_ protofibril exposed samples (D). The flotillin-1 expression remained stable from day 0–2 to day 2–5 (*p* = 0.44) (E). The flotillin-1 intensity relative to control was 1.1±0.6 in day 0–2 samples and 1.2±0.4 in day 2–5 samples (E). These data illustrate that the vesicular release remained unchanged, whereas the protein expression profile differed in response to Aβ_42_ protofibrils. Ultracentrifugation with two different gravitational forces was performed in an attempt to separate larger MVs and exosomes. Interestingly, most of the the apoE was found in the exosome fraction and was more prominent in EVs derived from Aβ_42_ protofibril treated cells, compared to control cells (F). Six independent cell culture experiments were analyzed for each of the two proteins apoE and flotillin-1 and examined with Wilcoxon matched-pairs signed rank test in C and E, and Mann-Whitney *U*-test in B and D.

Next, apoE expression levels were studied by western blot analysis to confirm the results from the MS analysis. At both day 0–2 and day 2–5, there was more than a threefold increase of apoE, indicating that apoE increased robustly in EVs in response to Aβ_42_ protofibril exposure (*p* = 0.03 for both time points, Fig. 6A-B). The relative abundance of apoE to controls remained constant between day 0–2 and day 2–5 (*p* = 0.69, Fig. 6C). The total EV protein content from the six independent cell culture experiments showed no difference in protein concentration in Aβ_42_ protofibril exposed cultures compared to unexposed cultures (day 2: 0.5±0.1*μ*g/*μ*l and 0.5±0.2*μ*g/*μ*l, respectively; day 5: 0.5±0.1*μ*g/*μ*l and 0.5±0.1*μ*g/*μ*l, respectively). To further investigate if the total amount of EVs was affected by the Aβ_42_ protofibril exposure, we performed western blot analysis with antibodies against the vesicle markers flotillin-1, TSG101 (both ∼48 kDa), and CD9 (∼25 kDa) (Fig. 6A). The expression of flotillin-1 remained unchanged upon exposure to Aβ_42_ protofibrils, both at day 0–2 (*p* = 0.94) and at day 2–5 (*p* = 0.59, Fig. 6D). Moreover, the relative expression of flotillin-1 remained constant between day 0–2 and day 2–5 (*p* = 0.44, Fig. 6E). Also TSG101 and CD9 remained stable over time and did not change between the two treatment groups (Fig. 6A), supporting that the changes observed in the vesicular protein content was not a result of differences in the vesicle release. In attempt to discriminate between exosomes and larger MVs, we performed experiments in which the media samples were first ultracentrifuged at 10,000 *x g* (to pellet MVs), followed by a second ultracentrifugation of the supernatant at 135,000 *x g* (to pellet exosomes). Interestingly, western blot analysis of the exosome and MV lysates, derived from Aβ_42_ protofibril exposed and untreated cultures, revealed that the majority of the apoE was situated in exosomes, rather than in larger MVs (Fig. 6F).

### Apolipoprotein E is primarily situated inside isolated EVs

**Fig.7:**
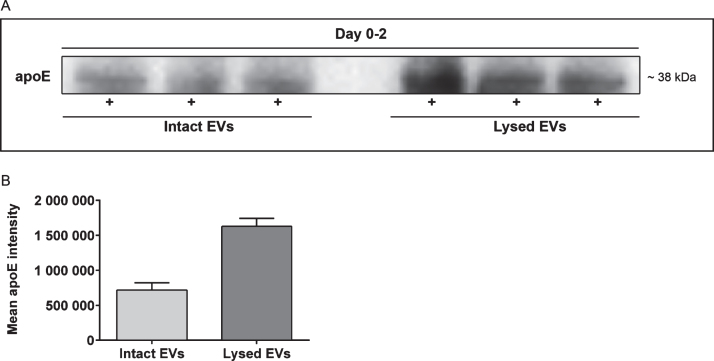
Apolipoprotein E is primarily situated inside isolated EVs. Immunoprecipitation was performed to confirm the presence of apoE inside the EVs. Following ultracentrifugation, EVs were either resuspended in PBS (intact EVs) or lysis buffer with protease inhibitors (lysed EVs) and immunoprecipitated with apoE antibodies. Western blot analysis from day 0–2 revealed twice as much apoE in the lysed EV samples, compared to the samples with intact EVs (A). The mean apoE intensity was 7.2E5±1.1E5 and 16.3E5±1.2E5 for intact EVs and lysed EVs, respectively (B). The data is from three independent cell culture experiments.

To confirm that apoE was in fact present inside the EVs, we performed parallel immunoprecipitations. Following ultracentrifugation, the EVs were resuspended in PBS (to keep the EVs intact) or in lysis buffer with protease inhibitors (to lyse the EVs and expose their content). In order to compare the levels of apoE situated inside or outside the EVs, the samples were immunoprecipitated using antibodies specific to apoE. Western blot analysis of the immunoprecipitates revealed that most apoE was located inside the EVs (Fig. 7A). Intensity measurements of the western blot bands revealed almost twice as much apoE in the lysed EV pellet fraction compared to the intact EV fraction, confirming that the majority of apoE was situated inside the EVs (Fig. 7B). Analysis of the non-bound fraction after immunoprecipitation showed no difference in apoE levels (in the samples derived from the EVs resuspended in PBS versus lysis buffer), indicating that there was an excess of apoE in relation to the immunoprecipitating antibody.

### Intracellular expression of apoE in Aβ_42_ protofibril exposed and unexposed cells

**Fig.8:**
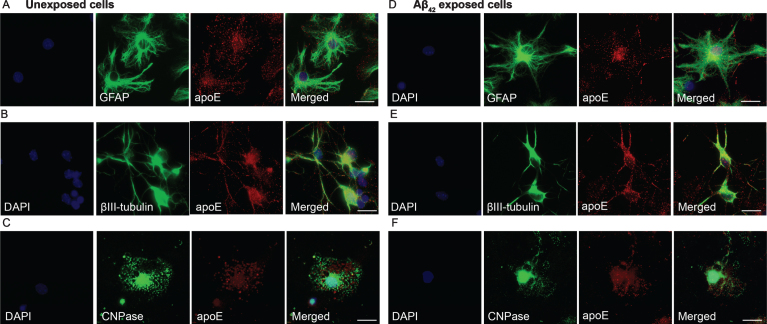
Immunostainings of apoE shows a similar intracellular expression pattern in Aβ_42_ protofibril exposed and unexposed cells. Astrocytes (A), neurons (B), and oligodendrocytes (C) from day 2 revealed that apoE was expressed in all three cell types, although to a greater extent in astrocytes and neurons than in oligodendrocytes. Following Aβ_42_ protofibril exposure, astrocytes displayed a high apoE expression around the nucleus, but a vesicular, dotted expression pattern was also found close to the plasma membrane, indicating a possible apoE secretion (D). Neurons showed a widespread expression of apoE throughout the cell soma and the dendrites (E). Some apoE expression was also observed in the few oligodendrocytes that were present in the culture (F), however in comparison to astrocytes and neurons, the expression was very low. DAPI (blue), GFAP, βIII-tubulin, CNPase (all green), and apoE (red). Scale bars: A-F = 20*μ*m.

**Fig.9:**
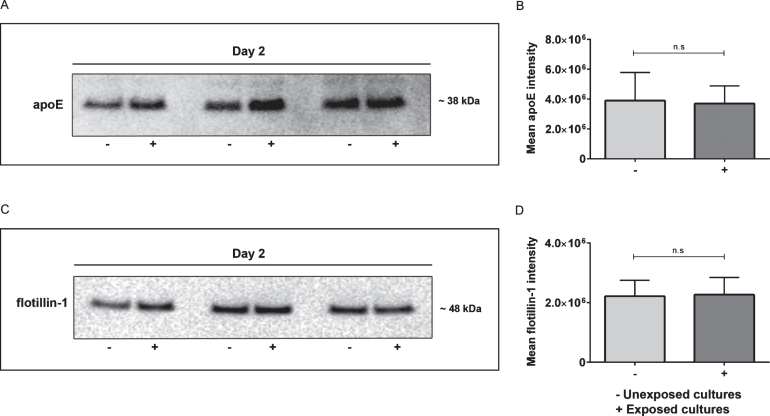
Intracellular apoE levels are similar in Aβ_42_ protofibril exposed cells and unexposed cells. Western blot analysis of cell lysates from day 2 verified that there was no difference in intracellular apoE expression in Aβ_42_ protofibril exposed cultures and unexposed cultures (A,B). The mean apoE intensity was 38.9E5±18.9E5 in unexposed samples and 37.0E5±11.9E5 in Aβ_42_ protofibril exposed samples. Flotillin-1 levels also remained unchanged in Aβ_42_ protofibril exposed cells, compared to untreated cells (C,D). The mean flotillin-1 intensity was 22.2E5±5.3E5 in unexposed samples and 22.7E5±5.8E5 in Aβ_42_ protofibril exposed samples. Six independent cell culture experiments were analyzed for each of the two proteins and the data was examined with Mann-Whitney *U*-test. Western blot analysis from three independent experiments are illustrated in A and C.

Having confirmed the high levels of apoE in EVs derived from Aβ_42_ protofibril exposed cultures by MS, western blot analysis, and immunoprecipitation, we next sought to investigate the apoE expression in our cell culture system using immunocytochemistry. Astrocytes, neurons, and oligodendrocytes were immunostained for GFAP, βIII-tubulin, and CNPase, respectively, in combination with anti-apoE specific antibodies. Apolipoprotein E was found to be expressed in all cell types, but more prominent in astrocytes (Fig. 8A) and neurons (Fig. 8B), compared to oligodendrocytes (Fig. 8C). Following Aβ_42_ protofibril exposure, astrocytes showed a robust presence of apoE around the nuclei, but also extensive staining of puncta at the plasma membrane, possibly indicating the presence of apoE in vesicles bound for secretion (Fig. 8D). Furthermore, Aβ_42_ protofibril exposed neurons showed a high expression of apoE throughout both the cell soma and the dendrites (Fig. 8E). Oligodendrocytes displayed a similar pattern of apoE expression in Aβ_42_ protofibril exposed cultures and in unexposed cultures (Fig. 8F). In an attempt to study the subcellular co-localization of apoE, we performed co-stainings with the vesicular marker CD9 and the trans-golgi subcellular marker GM-160. No clear co-localization was observed between apoE and these markers, and the expression pattern of CD9 or GM-160 were not altered by the Aβ_42_ protofibril treatment (Supplementary Figure 2). To further investigate the intracellular presence of apoE, we performed western blot analysis of total cell lysates. Immunoreactive western blot bands were clearly visible at ∼38 kDa for apoE (Fig. 9A) and at ∼48 kDa for flotillin-1 (Fig. 9B). The apoE levels did not differ between Aβ_42_ protofibril exposed cultures and unexposed cultures (Fig. 9C) and also the expression of flotillin-1 remained stable (Fig. 9D). Taken together, these results demonstrate that although Aβ_42_ protofibril exposure results in a prominent increase of apoE in EVs, the intracellular expression of the protein is unchanged.

## DISCUSSION

A vast number of studies suggest a central role of EVs in different diseases, making them an appealing treatment target. The data regarding EVs contribution to AD pathology is, however, complex. On one hand, it has been demonstrated that EVs can spread toxic forms of Aβ and hyperphosphorylated tau between cells and thereby contribute to neuronal loss [16, 26, 41, 42]. On the other hand, EVs are known to transfer neuroprotective substances between cells and have been suggested to reduce the Aβ load through microglial uptake [43, 44]. Regardless of their detrimental and protective effects, EVs constitute a promising source for biomarkers, due to the fact that they mirror the intracellular status of the cell of origin [29].

By using a co-culture system of neurons and glia, we here demonstrate that apoE is released and significantly increased in EVs in response to Aβ_42_ protofibril exposure. Notably, the release of EVs did not vary in response to Aβ_42_ protofibril exposure, although the protein content in the EVs changed markedly. Immunoprecipitation studies of intact EVs (resuspended in PBS) and lysed EVs (resuspended in lysis buffer) confirmed the intravesicular location of apoE. Some apoE was, however, detected in the PBS-resuspended EV samples, originating from damaged EVs (as a result of the ultracentrifugation), apoE bound to the vesicle membrane, or apoE present outside the EVs. Previous studies suggest that Aβ has the potential to solubilize or permeabilize membranes through its lipid-binding properties [45, 46]. It has, however, been shown that Aβ do not disrupt membranes of isolated synaptic vesicles [47], but the effect of Aβ protofibrils on EV membranes remain unclear. Interestingly, we found that the apoE content in the total cell lysates were unchanged in cultures treated with Aβ_42_ protofibrils, compared to untreated controls, indicating that although the apoE secretion was increased, the intracellular depots remained stable. Moreover, we found that the majority of the apoE was situated in exosomes, rather than in larger MVs, indicating an endosomal origin [48].

We have previously demonstrated that the astrocytes in the co-culture engulf and accumulate large amounts of Aβ_42_ protofibrils. The intracellular storage of Aβ results in severe astrocytic endosomal/lysosomal defects and consequently secretion of EVs with neurotoxic content [16]. Our initial studies showed that the EVs contained aggregated, partly N-terminally truncated Aβ_42_ and that they induced secondary neuronal apoptosis, 6–12 days following Aβ_42_ protofibril exposure [16]. Based on these results, we selected day 0–2 and day 2–5 following Aβ_42_ protofibril addition as measurement time points in the present study, focusing on more detailed analysis of the EV content. The exposed cells were constantly incubated with Aβ_42_ protofibrils in order to maximize the EV content. Consistent with our previous data, the astrocytes in the culture contained large deposits of aggregated Aβ at both time points, but there were no signs of direct neuronal cell loss during the 5-day treatment period. These data indicate that the Aβ protofibril exposure had no direct toxic effect on the neurons in the culture and that the secondary neuronal cell death (that we noticed in our previous study) was not yet initiated at these time points.

The apoE *ɛ*4 allele is the strongest, known genetic risk factor for developing late-onset AD [49] and several investigations have focused on the role of apoE in Aβ pathology [50, 51]. It has been shown that apoE co-deposit with Aβ in the senile plaques [52], particularly in AD patients carrying apoE *ɛ*4 [53]. Moreover, it is known that apoE binds to the lipoprotein receptor-related protein 1 (LRP1) receptor that is involved in active outward transport of Aβ across the blood-brain barrier and is frequently found around senile plaques [54]. Previous studies have shown that apoE in the central nervous system (CNS) is primarily produced by astrocytes (and to some extent by microglia) and that it serves to transport cholesterol to neurons via apoE receptors [55, 56]. Neurons may, however, also produce apoE under neuropathological conditions [57]. Our immunocytochemistry data demonstrate that mainly astrocytes and neurons in the cultures expressed apoE and both could therefore be the source of the apoE-containing vesicles. However, astrocytes represent the majority of cells in our cell culture system and are known to be highly secretory. In a recent study, focusing on rat astrocytes, the authors identified a decrease in exosome release following treatment with very high Aβ_42_ concentrations (2*μ*M, which is 20-fold compared to our investigation). Although there was a reduction in exosome secretion in their cell culture system, the apoE levels were unchanged, indicating that the apoE content in the exosomes was relatively increased.

Larger MVs and exosomes produced by different CNS cell types, including neurons and astrocytes, have been shown to contain a variety of Aβ species and other AβPP cleaving products [16, 58–60]. In addition, Aβ is known to interact with surface molecules on the EVs [61–63] and mixing Aβ_40_ and Aβ_42_ with neuron-derived exosomes accelerate the fibril formation [43]. Hence, EV-related proteins and/or lipids may promote aggregation of soluble Aβ species to less toxic fibrils. An example of such a fibrillization is the formation of physiologically functional fibrils of the premelanosomal protein (PMEL) in pigmented cells [64]. It has been shown that fibrillization of PMEL takes place in the presence of apoE at the surface of intraluminal vesicles and thereby prevents production and spreading of cytotoxic soluble amyloid aggregates [65]. Interestingly, when vesicles fuse with the plasma membrane, the apoE-containing intraluminal vesicles are released as exosomes. We suggest that a similar “defense” mechanism may occur in the neuroglial co-culture system, although this needs to be further investigated.

In conclusion, our analysis of the EV content in a co-culture of neurons and glia identified apoE to be significantly increased in response to Aβ_42_ protofibril exposure. We have previously shown that the inability of astrocytes to clear Aβ leads to increased release of truncated forms of Aβ in EVs [16]. However, whether the secreted Aβ are situated in the same EVs as apoE and in which way the apoE in EVs promote or counteract the Aβ pathology remain to be investigated.

## Supplementary Material

Supplementary Figure 1 Lack of B27 supplement in medium does not reduce cell survival during the experiment. To investigate the impact of lack of B27 supplement in the media the Aβ_42_ protofibril exposed and unexposed cells from day 0, 2, and 5 following treatment were fixed and labeled with TUNEL and DAPI (A-D). Ten systematically taken images/coverslip were analyzed by manually quantifying the number of TUNEL positive cells and the number of living DAPI nuclei (non-condensed nuclei with visible chromatin and not stained for TUNEL). Two independent cell culture experiments were performed, demonstrating that the B27 does not have a negative effect on cell survival (E). Scale bars: A-D=20 μm.

Supplementary Figure 2 Co-staining of apolipoprotein E with the subcellular markers CD9 and GM-160. Cell cultures were stained with antibodies to apoE in combination with the vesicular marker CD9 or the trans-Golgi subcellular marker GM-160. No clear co-localization was observed between apoE and these markers and Aβ_42_ protofibril treatment did not alter the expression pattern of CD9 or GM-160.

Supplementary Table 1 Protein list of all the proteins found in the EVs. All 807 proteins found in the EVs are numbered and presented in alphabetical order.

Supplementary Table 2 Differentially expressed proteins in EVs following Aβ_42_ protofibril exposure. The MS analysis revealed 807 unique proteins, in which five were found to be significantly differentially expressed in Aβ_42_ protofibril exposed cultures compared to unexposed cultures. In particular, apoE stood out due to its significant increase at both time points. Data is from three independent cell cultures.
