# Supplementary figures and images for: Increased Release of Apolipoprotein E in Extracellular Vesicles Following Amyloid-β Protofibril Exposure of Neuroglial Co-Cultures
Source: J Alzheimers Dis. 2017 Aug 29;60(1):305–21. doi: 10.3233/JAD-170278 (PMC5676865; doi:10.3233/JAD-170278)

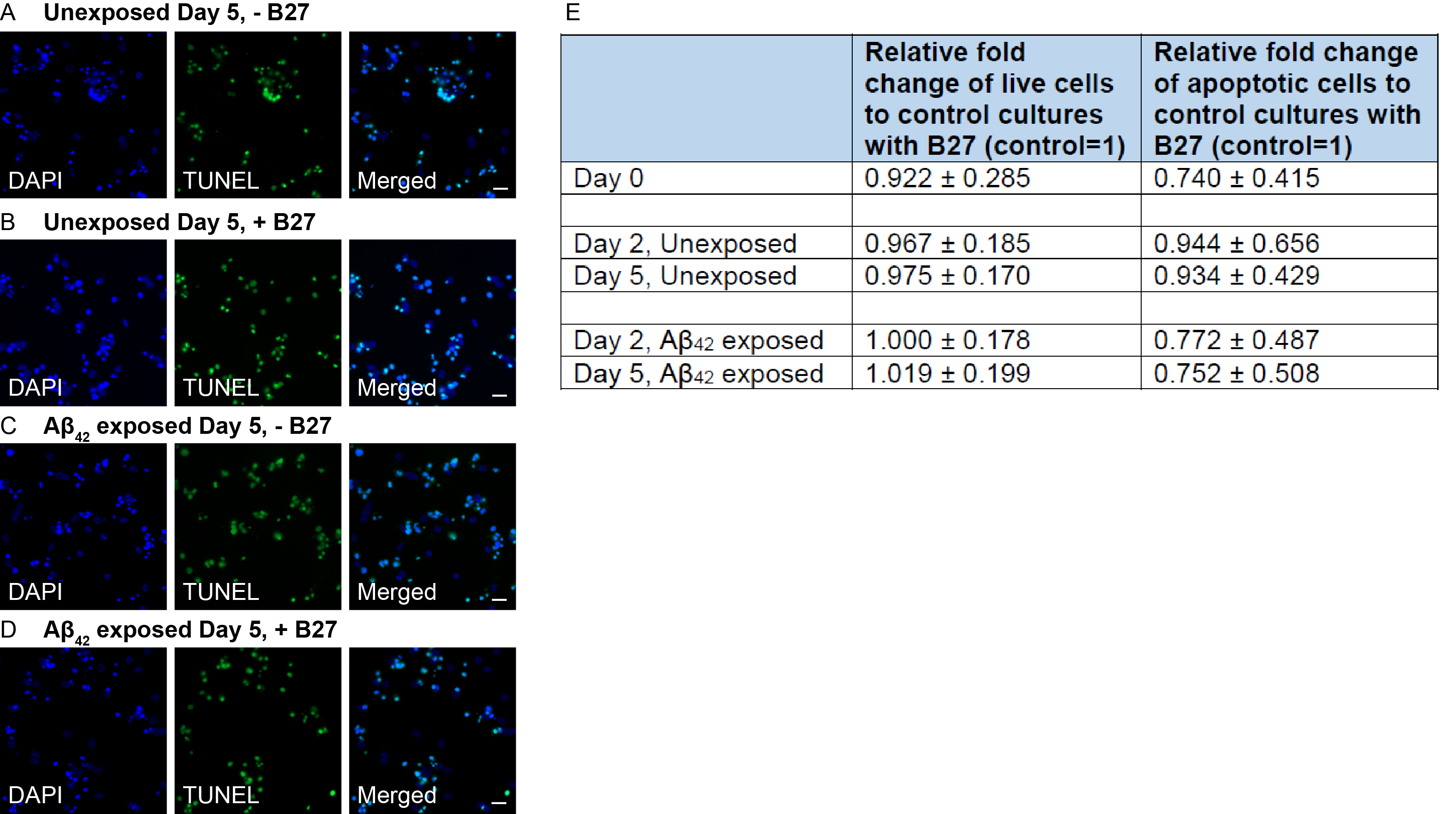

Supplement: Supplementary Figure 1 — Lack of B27 supplement in medium does not reduce cell survival during the experiment. To investigate the impact of lack of B27 supplement in the media the Aβ42 protofibril exposed and unexposed cells from day 0, 2, and 5 following treatment were fixed and labeled with TUNEL and DAPI (A-D). Ten systematically taken images/coverslip were analyzed by manually quantifying the number of TUNEL positive cells and the number of living DAPI nuclei (non-condensed nuclei with visible chromatin and not stained for TUNEL). Two independent cell culture experiments were performed, demonstrating that the B27 does not have a negative effect on cell survival (E). Scale bars: A-D=20 μm. [file jad-60-jad170278-s001.tif]

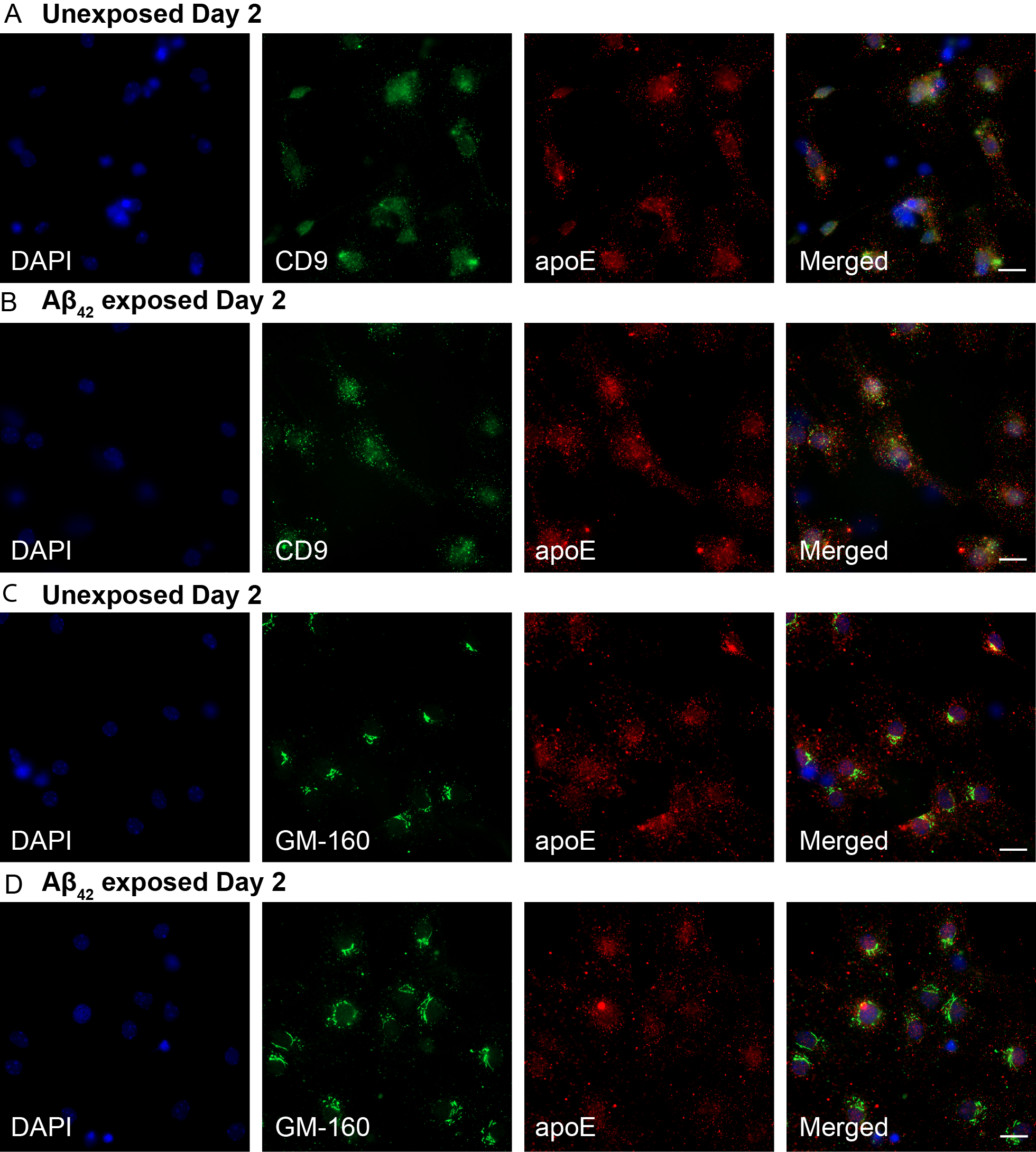

Supplement: Supplementary Figure 2 — Co-staining of apolipoprotein E with the subcellular markers CD9 and GM-160. Cell cultures were stained with antibodies to apoE in combination with the vesicular marker CD9 or the trans-Golgi subcellular marker GM-160. No clear co-localization was observed between apoE and these markers and Aβ42 protofibril treatment did not alter the expression pattern of CD9 or GM-160. [file jad-60-jad170278-s002.tif]

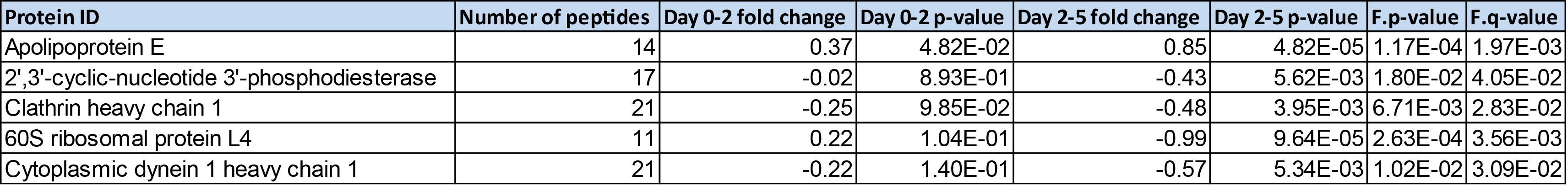

Supplement: Supplementary Table 2 — Differentially expressed proteins in EVs following Aβ42 protofibril exposure. The MS analysis revealed 807 unique proteins, in which five were found to be significantly differentially expressed in Aβ42 protofibril exposed cultures compared to unexposed cultures. In particular, apoE stood out due to its significant increase at both time points. Data is from three independent cell cultures. [file jad-60-jad170278-s004.tif]
