# Supplementary material for: Increased Release of Apolipoprotein E in Extracellular Vesicles Following Amyloid-β Protofibril Exposure of Neuroglial Co-Cultures
Source: J Alzheimers Dis. 2017 Aug 29;60(1):305–21. doi: 10.3233/JAD-170278 (PMC5676865; doi:10.3233/JAD-170278)
Supplement: Supplementary Table 1 — Protein list of all the proteins found in the EVs. All 807 proteins found in the EVs are numbered and presented in alphabetical order. [file jad-60-jad170278-s003.pdf]

	Protein name (alphabetical order)
1	10 kDa heat shock protein, mitochondrial (Hsp10) (10 kDa chaperonin) (Chaperonin 10) (CPN10)
2	14-3-3 protein beta/alpha (Protein kinase C inhibitor protein 1) (KCIP-1) [Cleaved into: 14-3-3 protein beta/alpha, N-terminally processed]
3	14-3-3 protein epsilon (14-3-3E)
4	14-3-3 protein gamma [Cleaved into: 14-3-3 protein gamma, N-terminally processed]
5	14-3-3 protein theta (14-3-3 protein tau)
6	14-3-3 protein zeta/delta (Protein kinase C inhibitor protein 1) (KCIP-1) (SEZ-2)
7	1-phosphatidylinositol 4,5-bisphosphate phosphodiesterase beta-2 (EC 3.1.4.11) (Phosphoinositide phospholipase C-beta-2) (Phospholipase C-beta-2) (PLC-beta-2)
8	1-phosphatidylinositol 4,5-bisphosphate phosphodiesterase delta-3 (EC 3.1.4.11) (Phosphoinositide phospholipase C-delta-3) (Phospholipase C-delta-3) (PLC-delta-3)
9	2',3'-cyclic-nucleotide 3'-phosphodiesterase (CNP) (CNPase) (EC 3.1.4.37)
10	26S protease regulatory subunit 10B (26S proteasome AAA-ATPase subunit RPT4) (Proteasome 26S subunit ATPase 6) (Proteasome subunit p42)
11	26S protease regulatory subunit 4 (P26s4) (26S proteasome AAA-ATPase subunit RPT2) (Proteasome 26S subunit ATPase 1)
12	26S protease regulatory subunit 6A (26S proteasome AAA-ATPase subunit RPT5) (Proteasome 26S subunit ATPase 3) (Tat-binding protein 1) (TBP-1)
13	26S protease regulatory subunit 7 (26S proteasome AAA-ATPase subunit RPT1) (Proteasome 26S subunit ATPase 2) (Protein MSS1)
14	26S protease regulatory subunit 8 (26S proteasome AAA-ATPase subunit RPT6) (Proteasome 26S subunit ATPase 5) (Proteasome subunit p45) (p45/SUG) (mSUG1)
15	26S proteasome non-ATPase regulatory subunit 1 (26S proteasome regulatory subunit RPN2) (26S proteasome regulatory subunit S1)
16	26S proteasome non-ATPase regulatory subunit 11 (26S proteasome regulatory subunit RPN6) (26S proteasome regulatory subunit S9) (26S proteasome regulatory subunit p44.5)
17	26S proteasome non-ATPase regulatory subunit 13 (26S proteasome regulatory subunit RPN9) (26S proteasome regulatory subunit S11) (26S proteasome regulatory subunit p40.5)
18	26S proteasome non-ATPase regulatory subunit 2 (26S proteasome regulatory subunit RPN1) (26S proteasome regulatory subunit S2) (26S proteasome subunit p97)
19	26S proteasome non-ATPase regulatory subunit 3 (26S proteasome regulatory subunit RPN3) (26S proteasome regulatory subunit S3) (Proteasome subunit p58) (Transplantation antigen P91A) (Tum-P91A antigen)
20	26S proteasome non-ATPase regulatory subunit 5 (26S protease subunit S5 basic) (26S proteasome subunit S5B)
21	26S proteasome non-ATPase regulatory subunit 7 (26S proteasome regulatory subunit RPN8) (26S proteasome regulatory subunit S12) (Mov34 protein) (Proteasome subunit p40)
22	2-hydroxyacylsphingosine 1-beta-galactosyltransferase (EC 2.4.1.47) (Ceramide UDP-galactosyltransferase) (Cerebroside synthase) (UDP-galactose-ceramide galactosyltransferase)
23	3-ketoacyl-CoA thiolase A, peroxisomal (EC 2.3.1.16) (Acetyl-CoA acyltransferase A) (Beta-ketothiolase A) (Peroxisomal 3-oxoacyl-CoA thiolase A)
24	40S ribosomal protein S10

25	40S ribosomal protein S11
26	40S ribosomal protein S12
27	40S ribosomal protein S13
28	40S ribosomal protein S14
29	40S ribosomal protein S15 (RIG protein)
30	40S ribosomal protein S16
31	40S ribosomal protein S17
32	40S ribosomal protein S18 (Ke-3) (Ke3)
33	40S ribosomal protein S19
34	40S ribosomal protein S2 (40S ribosomal protein S4) (Protein LLRep3)
35	40S ribosomal protein S20
36	40S ribosomal protein S23
37	40S ribosomal protein S24
38	40S ribosomal protein S25
39	40S ribosomal protein S26
40	40S ribosomal protein S27
41	40S ribosomal protein S3 (EC 4.2.99.18)
42	40S ribosomal protein S3a (Protein TU-11)
43	40S ribosomal protein S4, X isoform
44	40S ribosomal protein S5 [Cleaved into: 40S ribosomal protein S5, N-terminally processed]
45	40S ribosomal protein S6 (Phosphoprotein NP33)
46	40S ribosomal protein S7
47	40S ribosomal protein S8
48	40S ribosomal protein S9
49	40S ribosomal protein SA (37 kDa laminin receptor precursor) (37LRP) (37 kDa oncofetal antigen) (37/67 kDa laminin receptor) (LRP/LR) (67 kDa laminin receptor) (67LR) (Laminin receptor 1) (LamR) (Laminin-binding protein precursor p40) (LBP/p40) (OFA/iLRP)
50	60 kDa heat shock protein, mitochondrial (EC 3.6.4.9) (60 kDa chaperonin) (Chaperonin 60) (CPN60) (HSP-65) (Heat shock protein 60) (HSP-60) (Hsp60) (Mitochondrial matrix protein P1)
51	60S acidic ribosomal protein P0 (60S ribosomal protein L10E)
52	60S acidic ribosomal protein P1
53	60S acidic ribosomal protein P2
54	60S ribosomal protein L10a (CSA-19) (Neural precursor cell expressed developmentally down-regulated protein 6) (NEDD-6)
55	60S ribosomal protein L10-like/60S ribosomal protein L10
56	60S ribosomal protein L11
57	60S ribosomal protein L12
58	60S ribosomal protein L13 (A52)
59	60S ribosomal protein L13a (Transplantation antigen P198) (Tum-P198 antigen)
60	60S ribosomal protein L14
61	60S ribosomal protein L15
62	60S ribosomal protein L17
63	60S ribosomal protein L18

64	60S ribosomal protein L18a
65	60S ribosomal protein L19
66	60S ribosomal protein L21
67	60S ribosomal protein L22 (Heparin-binding protein HBp15)
68	60S ribosomal protein L23
69	60S ribosomal protein L23a
70	60S ribosomal protein L24
71	60S ribosomal protein L26 (Silica-induced gene 20 protein) (SIG-20)
72	60S ribosomal protein L27
73	60S ribosomal protein L27a (L29)
74	60S ribosomal protein L29
75	60S ribosomal protein L3 (J1 protein)
76	60S ribosomal protein L30
77	60S ribosomal protein L32
78	60S ribosomal protein L34
79	60S ribosomal protein L35
80	60S ribosomal protein L36
81	60S ribosomal protein L37a
82	60S ribosomal protein L39
83	60S ribosomal protein L4
84	60S ribosomal protein L5
85	60S ribosomal protein L6 (TAX-responsive enhancer element-binding protein 107) (TAXREB107)
86	60S ribosomal protein L7
87	60S ribosomal protein L7a (Surfeit locus protein 3)
88	60S ribosomal protein L8
89	60S ribosomal protein L9
90	78 kDa glucose-regulated protein (GRP-78) (Heat shock 70 kDa protein 5) (Immunoglobulin heavy chain-binding protein) (BiP)
91	A disintegrin and metalloproteinase with thrombospondin motifs 20 (ADAM-TS 20) (ADAM-TS20) (ADAMTS-20) (EC 3.4.24.-)
92	Acetyl-CoA acetyltransferase, mitochondrial (EC 2.3.1.9) (Acetoacetyl-CoA thiolase)
93	Acidic leucine-rich nuclear phosphoprotein 32 family member A (Acidic nuclear phosphoprotein pp32) (Leucine-rich acidic nuclear protein) (LANP) (Potent heat-stable protein phosphatase 2A inhibitor I1PP2A)
94	Acidic leucine-rich nuclear phosphoprotein 32 family member B (Proliferation-related acidic leucine-rich protein PAL31)
95	Acidic leucine-rich nuclear phosphoprotein 32 family member E (Cerebellar postnatal development protein 1) (LANP-like protein) (LANP-L)
96	Aconitate hydratase, mitochondrial (Aconitase) (EC 4.2.1.3) (Citrate hydro-lyase)
97	Actin, cytoplasmic 1/Actin, cytoplasmic 2
98	Actin-related protein 2 (Actin-like protein 2)
99	Actin-related protein 2/3 complex subunit 3 (Arp2/3 complex 21 kDa subunit) (p21-ARC)

100	Actin-related protein 2/3 complex subunit 4 (Arp2/3 complex 20 kDa subunit) (p20-ARC)
101	Activating transcription factor 7-interacting protein 1 (ATFa-associated modulator) (mAM) (MBD1-containing chromatin-associated factor 1)
102	Acyl-CoA-binding protein (ACBP) (Diazepam-binding inhibitor) (DBI) (Endozepine) (EP)
103	Acyl-coenzyme A thioesterase THEM4 (Acyl-CoA thioesterase THEM4) (EC 3.1.2.2) (Carboxyl-terminal modulator protein) (Thioesterase superfamily member 4)
104	Adenosylhomocysteinase (AdoHcyase) (EC 3.3.1.1) (CUBP) (Liver copper-binding protein) (S-adenosyl-L-homocysteine hydrolase)
105	ADP/ATP translocase 1 (ADP,ATP carrier protein 1) (ADP,ATP carrier protein, heart/skeletal muscle isoform T1) (Adenine nucleotide translocator 1) (ANT 1) (Solute carrier family 25 member 4) (mANC1)
106	ADP/ATP translocase 2 (ADP,ATP carrier protein 2) (Adenine nucleotide translocator 2) (ANT 2) (Solute carrier family 25 member 5) [Cleaved into: ADP/ATP translocase 2, N-terminally processed]
107	ADP-ribosylation factor 4
108	ADP-ribosylation factor-like protein 3
109	ADP-ribosylation factor-like protein 8A (ADP-ribosylation factor-like protein 10B) (Novel small G protein indispensable for equal chromosome segregation 2)
110	Alcohol dehydrogenase class-3 (EC 1.1.1.1) (Alcohol dehydrogenase 2) (Alcohol dehydrogenase 5) (Alcohol dehydrogenase B2) (ADH-B2) (Alcohol dehydrogenase class-III) (Glutathione-dependent formaldehyde dehydrogenase) (FALDH) (FDH) (GSH-FDH) (EC 1.1.1.-) (S-(hydroxymethyl)glutathione dehydrogenase) (EC 1.1.1.284)
111	Alpha-1,3/1,6-mannosyltransferase ALG2 (EC 2.4.1.132) (EC 2.4.1.257) (Asparagine-linked glycosylation protein 2 homolog) (GDP-Man:Man(1)GlcNAc(2)-PP-Dol alpha-1,3-mannosyltransferase) (GDP-Man:Man(1)GlcNAc(2)-PP-dolichol mannosyltransferase) (GDP-Man:Man(2)GlcNAc(2)-PP-Dol alpha-1,6-mannosyltransferase)
112	Alpha-2-macroglobulin-P (Alpha-2-macroglobulin)
113	Alpha-adducin (Erythrocyte adducin subunit alpha)
114	Alpha-centractin (Centractin) (ARP1) (Actin-RPV) (Centrosome-associated actin homolog)
115	Alpha-enolase (EC 4.2.1.11) (2-phospho-D-glycerate hydro-lyase) (Enolase 1) (Non-neural enolase) (NNE)
116	Alpha-mannosidase 2C1 (EC 3.2.1.24) (Alpha-D-mannoside mannohydrolase) (Mannosidase alpha class 2C member 1)
117	Alpha-N-acetyl-neuraminyl-2,3-beta-galactosyl-1,3-N-acetyl-galactosaminide alpha-2,6-sialyltransferase (EC 2.4.99.7) (NeuAc-alpha-2,3-Gal-beta-1,3-GalNAc-alpha-2,6-sialyltransferase) (ST6GalNAc IV) (ST6GalNAcIV) (Sialyltransferase 7D) (SIAT7-D)
118	Alpha-protein kinase 1 (EC 2.7.11.-) (Lymphocyte alpha-protein kinase)
119	Alpha-taxilin
120	Amyloid beta A4 protein (ABPP) (APP) (Alzheimer disease amyloid A4 protein homolog) (Amyloid precursor protein) (Amyloidogenic glycoprotein) (AG) (Beta-amyloid precursor protein) [Cleaved into: N-APP; Soluble APP-alpha (S-APP-alpha); Soluble APP-beta (S-APP-beta); C99 (APP-C99); Beta-amyloid protein 42 (Beta-APP42); Beta-amyloid protein 40 (Beta-APP40); C83; P3(42); P3(40); C80; Gamma-

	secretase C-terminal fragment 59 (APP-C59) (Amyloid intracellular domain 59) (AID(59)) (Gamma-CTF(59)); Gamma-secretase C-terminal fragment 57 (APP-C57) (Amyloid intracellular domain 57) (AID(57)) (Gamma-CTF(57)); Gamma-secretase C-terminal fragment 50 (Amyloid intracellular domain 50) (AID(50)) (Gamma-CTF(50)); C31]
121	Angiopoietin-2 (ANG-2)
122	Angiopoietin-like protein 8
123	Ankyrin repeat and MYND domain-containing protein 1
124	Ankyrin repeat and SAM domain-containing protein 1A (Odin)
125	Apolipoprotein E (Apo-E)
126	Apoptosis-stimulating of p53 protein 2 (Tumor suppressor p53-binding protein 2) (53BP2) (p53-binding protein 2) (p53BP2)
127	Arf-GAP with SH3 domain, ANK repeat and PH domain-containing protein 2 (Development and differentiation-enhancing factor 2) (Paxillin-associated protein with ARF GAP activity 3) (PAG3) (Pyk2 C-terminus-associated protein) (PAP)
128	Aryl hydrocarbon receptor nuclear translocator (ARNT protein) (Dioxin receptor, nuclear translocator) (Hypoxia-inducible factor 1-beta) (HIF-1-beta) (HIF1-beta)
129	Asparagine--tRNA ligase, cytoplasmic (EC 6.1.1.22) (Asparaginyl-tRNA synthetase) (AsnRS)
130	Aspartate aminotransferase, mitochondrial (mAspAT) (EC 2.6.1.1) (EC 2.6.1.7) (Fatty acid-binding protein) (FABP-1) (Glutamate oxaloacetate transaminase 2) (Kynurenine aminotransferase 4) (Kynurenine aminotransferase IV) (Kynurenine--oxoglutarate transaminase 4) (Kynurenine--oxoglutarate transaminase IV) (Plasma membrane-associated fatty acid-binding protein) (FABPpm) (Transaminase A)
131	Aspartate--tRNA ligase, cytoplasmic (EC 6.1.1.12) (Aspartyl-tRNA synthetase) (AspRS)
132	Astrocytic phosphoprotein PEA-15 (15 kDa phosphoprotein enriched in astrocytes)
133	ATP synthase F(0) complex subunit B1, mitochondrial (ATP synthase subunit b) (ATPase subunit b)
134	ATP synthase protein 8 (A6L) (F-ATPase subunit 8)
135	ATP synthase subunit alpha, mitochondrial
136	ATP synthase subunit beta, mitochondrial (EC 3.6.3.14)
137	ATP synthase subunit d, mitochondrial (ATPase subunit d)
138	ATP synthase subunit delta, mitochondrial (F-ATPase delta subunit)
139	ATP synthase subunit e, mitochondrial (ATPase subunit e)
140	ATP synthase subunit gamma, mitochondrial (F-ATPase gamma subunit)
141	ATP synthase subunit O, mitochondrial (Oligomycin sensitivity conferral protein) (OSCP)
142	ATP synthase-coupling factor 6, mitochondrial (ATPase subunit F6)
143	ATPase Asna1 (EC 3.6.-.-) (Arsenical pump-driving ATPase) (Arsenite-stimulated ATPase)
144	ATP-binding cassette sub-family B member 7, mitochondrial (ATP-binding cassette transporter 7) (ABC transporter 7 protein)
145	ATP-dependent RNA helicase A (RHA) (EC 3.6.4.13) (DEAH box protein 9) (mHEL-5) (Nuclear DNA helicase II) (NDH II)
146	ATP-dependent RNA helicase DDX1 (EC 3.6.4.13) (DEAD box protein 1)
147	AT-rich interactive domain-containing protein 3A (ARID domain-containing protein 3A) (B-cell regulator of IgH transcription) (Bright) (Dead ringer-like protein 1)

148	Aurora kinase A (EC 2.7.11.1) (Aurora 2) (Aurora family kinase 1) (Aurora/IPL1-related kinase 1) (ARK-1) (Aurora-related kinase 1) (Ipl1- and aurora-related kinase 1) (Serine/threonine-protein kinase 6) (Serine/threonine-protein kinase Ayk1) (Serine/threonine-protein kinase aurora-A)
149	Aurora kinase A and ninein-interacting protein
150	Band 4.1-like protein 2 (Generally expressed protein 4.1) (4.1G)
151	Basic leucine zipper and W2 domain-containing protein 2
152	Basigin (Basic immunoglobulin superfamily) (HT7 antigen) (Membrane glycoprotein gp42) (CD antigen CD147)
153	B-cell receptor CD22 (B-lymphocyte cell adhesion molecule) (BL-CAM) (Sialic acid-binding Ig-like lectin 2) (Siglec-2) (T-cell surface antigen Leu-14) (CD antigen CD22)
154	Bcl-2-binding component 3 (p53 up-regulated modulator of apoptosis)
155	Beta-2-syntrophin (59 kDa dystrophin-associated protein A1 basic component 2) (Syntrophin-3) (SNT3) (Syntrophin-like) (SNTL)
156	Beta-actin-like protein 2 (Kappa-actin)
157	Beta-centractin (Actin-related protein 1B) (ARP1B)
158	Beta-enolase (EC 4.2.1.11) (2-phospho-D-glycerate hydro-lyase) (Enolase 3) (Muscle-specific enolase) (MSE) (Skeletal muscle enolase)
159	BICD family-like cargo adapter 2 (Bicaudal D-related protein 2) (BICD-related protein 2) (BICDR-2) (Coiled-coil domain-containing protein 64B)
160	Bifunctional glutamate/proline--tRNA ligase (Bifunctional aminoacyl-tRNA synthetase) [Includes: Glutamate--tRNA ligase (EC 6.1.1.17) (Glutamyl-tRNA synthetase) (GluRS); Proline--tRNA ligase (EC 6.1.1.15) (Prolyl-tRNA synthetase) (ProRS)]
161	Brain acid soluble protein 1 (22 kDa neuronal tissue-enriched acidic protein) (Neuronal axonal membrane protein NAP-22)
162	Breast carcinoma-amplified sequence 1 homolog (Novel amplified in breast cancer 1 homolog)
163	Bromodomain adjacent to zinc finger domain protein 2A (Transcription termination factor I-interacting protein 5) (TTF-I-interacting protein 5) (Tip5)
164	Bromodomain-containing protein 2 (Female sterile homeotic-related protein 1) (Fsrg-1) (Protein RING3)
165	BUD13 homolog
166	Ca(2+)-independent N-acyltransferase (iNAT) (EC 2.3.1.-) (H-rev107-like protein 5) (HRAS-like suppressor 5) (HRSL5)
167	Calcium-activated chloride channel regulator 2 (EC 3.4.-.-) (Calcium-activated chloride channel family member 5) (mCLCA5)
168	Calcium-independent phospholipase A2-gamma (EC 3.1.1.5) (Intracellular membrane-associated calcium-independent phospholipase A2 gamma) (iPLA2-gamma) (Patatin-like phospholipase domain-containing protein 8)
169	Calmodulin
170	Calnexin
171	Calponin-3 (Calponin, acidic isoform)
172	Calreticulin (CRP55) (Calregulin) (Endoplasmic reticulum resident protein 60) (ERp60) (HACBP)
173	Caprin-1 (Cytoplasmic activation- and proliferation-associated protein 1) (GPI-anchored membrane protein 1) (GPI-anchored protein p137) (GPI-p137) (p137GPI)

	(Membrane component chromosome 11 surface marker 1) (RNA granule protein 105)
174	Carboxypeptidase E (CPE) (EC 3.4.17.10) (Carboxypeptidase H) (CPH) (Enkephalin convertase) (Prohormone-processing carboxypeptidase)
175	Carboxypeptidase N catalytic chain (CPN) (EC 3.4.17.3) (Carboxypeptidase N polypeptide 1) (Carboxypeptidase N small subunit)
176	Caseinolytic peptidase B protein homolog (EC 3.6.1.3) (Suppressor of potassium transport defect 3)
177	Catalase (EC 1.11.1.6)
178	Cathepsin D (EC 3.4.23.5)
179	Cathepsin L1 (EC 3.4.22.15) (Cathepsin L) (Major excreted protein) (MEP) (p39 cysteine proteinase) [Cleaved into: Cathepsin L1 heavy chain; Cathepsin L1 light chain]
180	CD63 antigen (CD antigen CD63)
181	CD81 antigen (26 kDa cell surface protein TAPA-1) (Target of the antiproliferative antibody 1) (CD antigen CD81)
182	CD9 antigen (CD antigen CD9)
183	CDGSH iron-sulfur domain-containing protein 1 (MitoNEET)
184	Cell cycle and apoptosis regulator protein 2 (Cell division cycle and apoptosis regulator protein 2)
185	Cell death activator CIDE-A (Cell death-inducing DFFA-like effector A)
186	Cell division control protein 42 homolog (G25K GTP-binding protein)
187	Cell division cycle protein 20 homolog (mmCdc20) (p55CDC)
188	Centriolin (Centrosomal protein 1) (Centrosomal protein of 110 kDa) (Cep110)
189	Centromere protein J (CENP-J)
190	Centromere-associated protein E (Centromere protein E) (CENP-E) (Kinesin superfamily protein 10) (KIF10) (Motor domain of KIF10)
191	Chloride intracellular channel protein 4 (mc3s5/mtCLIC)
192	Choline transporter-like protein 1 (Solute carrier family 44 member 1) (CD antigen CD92)
193	Chromobox protein homolog 3 (Heterochromatin protein 1 homolog gamma) (HP1 gamma) (M32) (Modifier 2 protein)
194	Chromobox protein homolog 5 (Heterochromatin protein 1 homolog alpha) (HP1 alpha)
195	Chromodomain-helicase-DNA-binding protein 2 (CHD-2) (EC 3.6.4.12) (ATP-dependent helicase CHD2)
196	Cilia- and flagella-associated protein 57 (WD repeat-containing protein 65)
197	Citrate synthase, mitochondrial (EC 2.3.3.1) (Citrate (Si)-synthase)
198	Clathrin heavy chain 1
199	Clusterin (Apolipoprotein J) (Apo-J) (Clustrin) (Sulfated glycoprotein 2) (SGP-2) [Cleaved into: Clusterin beta chain; Clusterin alpha chain]
200	Coatomer subunit gamma-1 (Gamma-1-coat protein) (Gamma-1-COP)
201	Cofilin-1 (Cofilin, non-muscle isoform)
202	Coiled-coil and C2 domain-containing protein 1A (Five prime repressor element under dual repression-binding protein 1) (FRE under dual repression-binding protein 1) (Freud-1)
203	Coiled-coil domain-containing protein 113

204	Coiled-coil domain-containing protein 115 (Coiled-coil protein 1) (Ccp1)
205	Coiled-coil domain-containing protein 177
206	Coiled-coil domain-containing protein 190
207	Coiled-coil domain-containing protein 39
208	Coiled-coil domain-containing protein 66
209	Coiled-coil domain-containing protein 73
210	Coiled-coil domain-containing protein 9
211	Coiled-coil domain-containing protein 91 (GGA-binding partner)
212	Coiled-coil domain-containing protein 96
213	Cold-inducible RNA-binding protein (A18 hnRNP) (Glycine-rich RNA-binding protein CIRP)
214	Collagen alpha-1(XIX) chain (Collagen alpha-1(Y) chain)
215	Collagen alpha-1(XVIII) chain [Cleaved into: Endostatin]
216	Contactin-1 (Neural cell surface protein F3)
217	COP9 signalosome complex subunit 4 (SGN4) (Signalosome subunit 4) (JAB1-containing signalosome subunit 4)
218	Core histone macro-H2A.1 (Histone macroH2A1) (mH2A1) (H2A.y) (H2A/y)
219	Core histone macro-H2A.2 (Histone macroH2A2) (mH2A2)
220	Coronin-2B
221	Creatine kinase B-type (EC 2.7.3.2) (B-CK) (Creatine kinase B chain)
222	Creatine kinase M-type (EC 2.7.3.2) (Creatine kinase M chain) (M-CK)
223	Crk-like protein
224	Cullin-3 (CUL-3)
225	Cullin-associated NEDD8-dissociated protein 1 (Cullin-associated and neddylation-dissociated protein 1) (p120 CAND1)
226	Cyclin-H
227	Cyclin-Y (Cyclin fold protein 1)
228	Cystatin-C (Cystatin-3)
229	Cysteine protease ATG4A (EC 3.4.22.-) (AUT-like 2 cysteine endopeptidase) (Autophagin-2) (Autophagy-related cysteine endopeptidase 2) (Autophagy-related protein 4 homolog A)
230	Cytochrome b-c1 complex subunit 1, mitochondrial (Complex III subunit 1) (Core protein I) (Ubiquinol-cytochrome-c reductase complex core protein 1)
231	Cytochrome b-c1 complex subunit 2, mitochondrial (Complex III subunit 2) (Core protein II) (Ubiquinol-cytochrome-c reductase complex core protein 2)
232	Cytochrome c oxidase subunit 2 (Cytochrome c oxidase polypeptide II)
233	Cytochrome c oxidase subunit 4 isoform 1, mitochondrial (Cytochrome c oxidase polypeptide IV) (Cytochrome c oxidase subunit IV isoform 1) (COX IV-1)
234	Cytochrome c oxidase subunit 5A, mitochondrial (Cytochrome c oxidase polypeptide Va)
235	Cytochrome c oxidase subunit 5B, mitochondrial (Cytochrome c oxidase polypeptide Vb)
236	Cytochrome c oxidase subunit NDUFA4
237	Cytochrome c1, heme protein, mitochondrial (Complex III subunit 4) (Complex III subunit IV) (Cytochrome b-c1 complex subunit 4) (Ubiquinol-cytochrome-c reductase complex cytochrome c1 subunit) (Cytochrome c-1)

238	Cytochrome P450 2A12 (EC 1.14.14.1) (CYPIIA12) (Steroid hormones 7-alpha-hydroxylase) (Testosterone 7-alpha-hydroxylase)
239	Cytokine-dependent hematopoietic cell linker (Mast cell immunoreceptor signal transducer)
240	Cytoplasmic dynein 1 heavy chain 1 (Cytoplasmic dynein heavy chain 1) (Dynein heavy chain, cytosolic)
241	Cytoplasmic dynein 1 light intermediate chain 2/Cytoplasmic dynein 1 light intermediate chain 1
242	D-3-phosphoglycerate dehydrogenase (3-PGDH) (EC 1.1.1.95) (A10)
243	D-beta-hydroxybutyrate dehydrogenase, mitochondrial (EC 1.1.1.30) (3-hydroxybutyrate dehydrogenase) (BDH)
244	Dedicator of cytokinesis protein 7 (Protein moonlight)
245	Dehydrogenase/reductase SDR family member 7B (EC 1.1.-.-) (Short-chain dehydrogenase/reductase family 32C member 1)
246	DENN domain-containing protein 2C
247	Destrin (Actin-depolymerizing factor) (ADF) (Sid 23)
248	Dihydrolipoyllysine-residue acetyltransferase component of pyruvate dehydrogenase complex, mitochondrial (EC 2.3.1.12) (Dihydrolipoamide acetyltransferase component of pyruvate dehydrogenase complex) (Pyruvate dehydrogenase complex component E2) (PDC-E2) (PDCE2)
249	Dihydropyrimidinase-related protein 1 (DRP-1) (Collapsin response mediator protein 1) (CRMP-1) (Unc-33-like phosphoprotein 3) (ULIP-3)
250	Dihydropyrimidinase-related protein 2 (DRP-2) (Unc-33-like phosphoprotein 2) (ULIP-2)
251	Dihydropyrimidinase-related protein 3 (DRP-3) (Unc-33-like phosphoprotein 1) (ULIP-1)
252	Dihydropyrimidinase-related protein 5 (DRP-5) (Collapsin response mediator protein 5) (CRMP-5)
253	Disco-interacting protein 2 homolog B (DIP2 homolog B)
254	DNA damage-binding protein 1 (DDB p127 subunit) (Damage-specific DNA-binding protein 1) (UV-damaged DNA-binding factor)
255	DNA damage-inducible transcript 3 protein (DDIT-3) (C/EBP zeta) (C/EBP-homologous protein) (CHOP) (C/EBP-homologous protein 10) (CHOP-10) (CCAAT/enhancer-binding protein homologous protein) (Growth arrest and DNA-damage-inducible protein GADD153)
256	DNA polymerase eta (EC 2.7.7.7) (RAD30 homolog A) (Xeroderma pigmentosum variant type protein homolog)
257	DNA topoisomerase 1 (EC 5.99.1.2) (DNA topoisomerase I)
258	DNA topoisomerase 2-beta (EC 5.99.1.3) (DNA topoisomerase II, beta isozyme)
259	DNA-binding protein RFX2 (Regulatory factor X 2)
260	DnaJ homolog subfamily A member 1 (DnaJ protein homolog 2) (Heat shock 40 kDa protein 4) (Heat shock protein J2) (HSJ-2)
261	DnaJ homolog subfamily A member 2 (mDj3)
262	DnaJ homolog subfamily B member 11 (APOBEC1-binding protein 2) (ABBP-2) (ER-associated DNAJ) (ER-associated Hsp40 co-chaperone) (Endoplasmic reticulum DNA J domain-containing protein 3) (ER-resident protein ERdj3) (ERdj3) (ERj3p)

263	Dolichyl-diphosphooligosaccharide--protein glycosyltransferase subunit 2 (Dolichyl-diphosphooligosaccharide--protein glycosyltransferase 63 kDa subunit) (Ribophorin II) (RPN-II) (Ribophorin-2)
264	Dolichyl-diphosphooligosaccharide--protein glycosyltransferase subunit DAD1 (Oligosaccharyl transferase subunit DAD1) (Defender against cell death 1) (DAD-1)
265	Dual specificity protein phosphatase 19 (EC 3.1.3.16) (EC 3.1.3.48) (Protein phosphatase SKRP1) (Stress-activated protein kinase pathway-regulating phosphatase 1)
266	Dynactin subunit 2 (50 kDa dynein-associated polypeptide) (Dynactin complex 50 kDa subunit) (DCTN-50) (Growth cone membrane protein 23-48K) (GMP23-48K) (p50 dynamitin)
267	Dynein heavy chain 2, axonemal (Axonemal beta dynein heavy chain 2) (Ciliary dynein heavy chain 2)
268	Dynein light chain roadblock-type 1 (Dynein light chain 2A, cytoplasmic)
269	Dystrophin
270	E3 ubiquitin-protein ligase RNF138 (EC 2.3.2.27) (RING finger protein 138) (RING-type E3 ubiquitin transferase RNF138)
271	E3 ubiquitin-protein ligase RNF213 (EC 2.3.2.27) (EC 3.6.4.-) (Mysterin) (RING finger protein 213) (RING-type E3 ubiquitin transferase RNF213)
272	E3 ubiquitin-protein ligase ZNF598 (EC 2.3.2.27) (Zinc finger protein 598)
273	Ectonucleotide pyrophosphatase/phosphodiesterase family member 6 (E-NPP 6) (NPP-6) (EC 3.1.4.-) (EC 3.1.4.38) (Choline-specific glycerophosphodiester phosphodiesterase) (Glycerophosphocholine cholinephosphodiesterase) (GPC-Cpde)
274	EF-hand calcium-binding domain-containing protein 6 (DJ-1-binding protein) (DJBP)
275	EF-hand domain-containing family member B
276	ELAV-like protein 1 (Elav-like generic protein) (Hu-antigen R) (HuR) (MelG)
277	ELAV-like protein 3 (Hu-antigen C) (HuC)
278	Electron transfer flavoprotein subunit alpha, mitochondrial (Alpha-ETF)
279	Elongation factor 1-alpha 1 (EF-1-alpha-1) (Elongation factor Tu) (EF-Tu) (Eukaryotic elongation factor 1 A-1) (eEF1A-1)
280	Elongation factor 1-delta (EF-1-delta)
281	Elongation factor 1-gamma (EF-1-gamma) (eEF-1B gamma)
282	Elongation factor 2 (EF-2)
283	Elongation factor Tu, mitochondrial
284	Elongin-C (EloC) (Elongin 15 kDa subunit) (RNA polymerase II transcription factor SIII subunit C) (SIII p15) (Stromal membrane-associated protein SMAP1B homolog) (Transcription elongation factor B polypeptide 1)
285	Endoplasmin (94 kDa glucose-regulated protein) (GRP-94) (Endoplasmic reticulum resident protein 99) (ERp99) (Heat shock protein 90 kDa beta member 1) (Polymorphic tumor rejection antigen 1) (Tumor rejection antigen gp96)
286	Endothelin B receptor (ET-B) (ET-BR) (Endothelin receptor non-selective type)
287	Enoyl-CoA hydratase, mitochondrial (EC 4.2.1.17) (Enoyl-CoA hydratase 1) (Short-chain enoyl-CoA hydratase) (SCEH)
288	Envoplakin (210 kDa cornified envelope precursor protein) (p210)
289	Eukaryotic initiation factor 4A-I (eIF-4A-I) (eIF4A-I) (EC 3.6.4.13) (ATP-dependent RNA helicase eIF4A-1)

290	Eukaryotic initiation factor 4A-III (eIF-4A-III) (eIF4A-III) (EC 3.6.4.13) (ATP-dependent RNA helicase DDX48) (ATP-dependent RNA helicase eIF4A-3) (DEAD box protein 48) (Eukaryotic translation initiation factor 4A isoform 3) [Cleaved into: Eukaryotic initiation factor 4A-III, N-terminally processed]
291	Eukaryotic translation initiation factor 3 subunit A (eIF3a) (Centrosomin) (Eukaryotic translation initiation factor 3 subunit 10) (eIF-3-theta) (eIF3 p167) (eIF3 p180) (eIF3 p185) (p162)
292	Eukaryotic translation initiation factor 3 subunit D (eIF3d) (Eukaryotic translation initiation factor 3 subunit 7) (eIF-3-zeta) (eIF3 p66)
293	Eukaryotic translation initiation factor 5A-1 (eIF-5A-1) (eIF-5A1) (Eukaryotic initiation factor 5A isoform 1) (eIF-5A) (eIF-4D)
294	Excitatory amino acid transporter 1 (Glial high affinity glutamate transporter) (High-affinity neuronal glutamate transporter) (Sodium-dependent glutamate/aspartate transporter 1) (GLAST-1) (Solute carrier family 1 member 3)
295	Ezrin (Cytovillin) (Villin-2) (p81)
296	F-actin-capping protein subunit alpha-2 (CapZ alpha-2)
297	F-actin-capping protein subunit beta (CapZ beta)
298	Far upstream element-binding protein 1 (FBP) (FUSE-binding protein 1)
299	Fascin (Singed-like protein)
300	Fatty acid synthase (EC 2.3.1.85) [Includes: [Acyl-carrier-protein] S-acetyltransferase (EC 2.3.1.38); [Acyl-carrier-protein] S-malonyltransferase (EC 2.3.1.39); 3-oxoacyl-[acyl-carrier-protein] synthase (EC 2.3.1.41); 3-oxoacyl-[acyl-carrier-protein] reductase (EC 1.1.1.100); 3-hydroxyacyl-[acyl-carrier-protein] dehydratase (EC 4.2.1.59); Enoyl-[acyl-carrier-protein] reductase (EC 1.3.1.39); Oleoyl-[acyl-carrier-protein] hydrolase (EC 3.1.2.14)]
301	Fatty acid-binding protein, brain (Brain lipid-binding protein) (BLBP) (Brain-type fatty acid-binding protein) (B-FABP) (Fatty acid-binding protein 7)
302	Fatty acid-binding protein, epidermal (Epidermal-type fatty acid-binding protein) (E-FABP) (Fatty acid-binding protein 5) (Keratinocyte lipid-binding protein) (Psoriasis-associated fatty acid-binding protein homolog) (PA-FABP)
303	Fatty aldehyde dehydrogenase (EC 1.2.1.3) (Aldehyde dehydrogenase 3) (Aldehyde dehydrogenase family 3 member A2)
304	F-box only protein 16
305	F-box only protein 30 (Muscle ubiquitin ligase of SCF complex in atrophy-1) (MUSA1)
306	Ferritin light chain 1 (Ferritin L subunit 1)
307	Fibromodulin (FM) (Collagen-binding 59 kDa protein) (Keratan sulfate proteoglycan fibromodulin) (KSPG fibromodulin)
308	Fibrous sheath-interacting protein 2
309	Filamin-A-interacting protein 1 (FILIP)
310	Forkhead-associated domain-containing protein 1 (FHA domain-containing protein 1)
311	Frataxin, mitochondrial (Fxn) (EC 1.16.3.1) [Cleaved into: Frataxin intermediate form; Frataxin mature form]
312	Fructose-bisphosphate aldolase A (EC 4.1.2.13) (Aldolase 1) (Muscle-type aldolase)
313	Fructose-bisphosphate aldolase C (EC 4.1.2.13) (Aldolase 3) (Brain-type aldolase) (Scrapie-responsive protein 2) (Zebrin II)
314	G patch domain-containing protein 1
315	G protein-regulated inducer of neurite outgrowth 3 (GRIN3)

316	Ganglioside GM2 activator (Cerebroside sulfate activator protein) (GM2-AP) (Sphingolipid activator protein 3) (SAP-3)
317	Gap junction alpha-1 protein (Connexin-43) (Cx43) (Gap junction 43 kDa heart protein)
318	Glia-derived nexin (GDN) (Peptidase inhibitor 7) (PI-7) (Protease nexin 1) (PN-1) (Protease nexin I) (Serine protease-inhibitor 4) (Serpin E2)
319	Glucosylceramidase (EC 3.2.1.45) (Acid beta-glucosidase) (Beta-glucocerebrosidase) (D-glucosyl-N-acylsphingosine glucohydrolase)
320	Glutamate dehydrogenase 1, mitochondrial (GDH 1) (EC 1.4.1.3)
321	Glutamate receptor ionotropic, NMDA 1 (GluN1) (Glutamate [NMDA] receptor subunit zeta-1) (N-methyl-D-aspartate receptor subunit NR1) (NMD-R1)
322	Glutamine synthetase (GS) (EC 6.3.1.2) (Glutamate decarboxylase) (EC 4.1.1.15) (Glutamate--ammonia ligase)
323	Glutamine--fructose-6-phosphate aminotransferase [isomerizing] 2 (EC 2.6.1.16) (D-fructose-6-phosphate amidotransferase 2) (Glutamine:fructose-6-phosphate amidotransferase 2) (GFAT 2) (GFAT2) (Hexosephosphate aminotransferase 2)
324	Glutaredoxin-2, mitochondrial
325	Glutaredoxin-3 (PKC-interacting cousin of thioredoxin) (PICOT) (PKC-theta-interacting protein) (PKCq-interacting protein) (Thioredoxin-like protein 2)
326	Glutathione S-transferase Mu 1 (EC 2.5.1.18) (GST 1-1) (GST class-mu 1) (Glutathione S-transferase GT8.7) (pmGT10)
327	Glutathione S-transferase P 1 (Gst P1) (EC 2.5.1.18) (GST YF-YF) (GST class-pi) (GST-piB) (Preadipocyte growth factor)
328	Glyceraldehyde-3-phosphate dehydrogenase (GAPDH) (EC 1.2.1.12) (Peptidyl-cysteine S-nitrosylase GAPDH) (EC 2.6.99.-)
329	Glycogen phosphorylase, brain form (EC 2.4.1.1)
330	Glycogen synthase kinase-3 beta (GSK-3 beta) (EC 2.7.11.26) (Serine/threonine-protein kinase GSK3B) (EC 2.7.11.1)
331	Glycolipid transfer protein (GLTP)
332	Glypican-2 [Cleaved into: Secreted glypican-2]
333	GTP-binding nuclear protein Ran (GTPase Ran) (Ras-like protein TC4) (Ras-related nuclear protein)
334	Guanine nucleotide-binding protein G(i) subunit alpha-2 (Adenylate cyclase-inhibiting G alpha protein)
335	Guanine nucleotide-binding protein G(I)/G(S)/G(O) subunit gamma-12
336	Guanine nucleotide-binding protein G(I)/G(S)/G(O) subunit gamma-2 (G gamma-I)
337	Guanine nucleotide-binding protein G(I)/G(S)/G(T) subunit beta-1 (Transducin beta chain 1)
338	Guanine nucleotide-binding protein G(I)/G(S)/G(T) subunit beta-2 (G protein subunit beta-2) (Transducin beta chain 2)
339	Guanine nucleotide-binding protein G(o) subunit alpha
340	Guanine nucleotide-binding protein subunit alpha-12/Guanine nucleotide-binding protein subunit alpha-13
341	H-2 class II histocompatibility antigen, E-Q beta chain (E-W17)
342	Heat shock 70 kDa protein 12A
343	Heat shock 70 kDa protein 1-like (Heat shock 70 kDa protein 1L) (Heat shock 70 kDa-like protein 1) (Spermatid-specific heat shock protein 70)
344	Heat shock 70 kDa protein 4 (Heat shock 70-related protein APG-2)

345	Heat shock cognate 71 kDa protein (Heat shock 70 kDa protein 8)
346	Heat shock protein 75 kDa, mitochondrial (HSP 75) (TNFR-associated protein 1) (Tumor necrosis factor type 1 receptor-associated protein) (TRAP-1)
347	Heat shock protein HSP 90-alpha (Heat shock 86 kDa) (HSP 86) (HSP86) (Tumor-specific transplantation 86 kDa antigen) (TSTA)
348	Heat shock protein HSP 90-beta (Heat shock 84 kDa) (HSP 84) (HSP84) (Tumor-specific transplantation 84 kDa antigen) (TSTA)
349	Heat shock-related 70 kDa protein 2 (Heat shock protein 70.2)
350	Hephaestin-like protein 1 (EC 1.-.-.-)
351	Heterochromatin protein 1-binding protein 3
352	Heterogeneous nuclear ribonucleoprotein A/B (hnRNP A/B) (CArG-binding factor-A) (CBF-A)
353	Heterogeneous nuclear ribonucleoprotein A0 (hnRNP A0)
354	Heterogeneous nuclear ribonucleoprotein A1 (hnRNP A1) (HDP-1) (Helix-destabilizing protein) (Single-strand-binding protein) (Topoisomerase-inhibitor suppressed) (hnRNP core protein A1) [Cleaved into: Heterogeneous nuclear ribonucleoprotein A1, N-terminally processed]
355	Heterogeneous nuclear ribonucleoprotein A3 (hnRNP A3)
356	Heterogeneous nuclear ribonucleoprotein D0 (hnRNP D0) (AU-rich element RNA-binding protein 1)
357	Heterogeneous nuclear ribonucleoprotein D-like (hnRNP D-like) (hnRNP DL) (JKT41-binding protein)
358	Heterogeneous nuclear ribonucleoprotein F (hnRNP F) [Cleaved into: Heterogeneous nuclear ribonucleoprotein F, N-terminally processed]
359	Heterogeneous nuclear ribonucleoprotein H (hnRNP H) [Cleaved into: Heterogeneous nuclear ribonucleoprotein H, N-terminally processed]
360	Heterogeneous nuclear ribonucleoprotein H2 (hnRNP H2) (Heterogeneous nuclear ribonucleoprotein H') (hnRNP H') [Cleaved into: Heterogeneous nuclear ribonucleoprotein H2, N-terminally processed]
361	Heterogeneous nuclear ribonucleoprotein K (hnRNP K)
362	Heterogeneous nuclear ribonucleoprotein L (hnRNP L)
363	Heterogeneous nuclear ribonucleoprotein L-like
364	Heterogeneous nuclear ribonucleoprotein M (hnRNP M)
365	Heterogeneous nuclear ribonucleoprotein Q (hnRNP Q) (Glycine- and tyrosine-rich RNA-binding protein) (GRY-RBP) (NS1-associated protein 1) (Synaptotagmin-binding, cytoplasmic RNA-interacting protein) (pp68)
366	Heterogeneous nuclear ribonucleoprotein U (hnRNP U) (Scaffold attachment factor A) (SAF-A)
367	Heterogeneous nuclear ribonucleoprotein U-like protein 2 (MLF1-associated nuclear protein)
368	Heterogeneous nuclear ribonucleoproteins A2/B1 (hnRNP A2/B1)
369	Heterogeneous nuclear ribonucleoproteins C1/C2 (hnRNP C1/C2)
370	High mobility group protein B1 (High mobility group protein 1) (HMG-1)
371	High mobility group protein B2 (High mobility group protein 2) (HMG-2)
372	Highly divergent homeobox
373	Histamine H3 receptor (H3R) (HH3R)

374	Histidine triad nucleotide-binding protein 1 (EC 3.-.-.-) (Adenosine 5'-monophosphoramidase) (Protein kinase C inhibitor 1) (Protein kinase C-interacting protein 1) (PKCI-1)
375	Histone acetyltransferase p300/CREB-binding protein
376	Histone acetyltransferase type B catalytic subunit (EC 2.3.1.48) (Histone acetyltransferase 1)
377	Histone deacetylase 1/Histone deacetylase 2
378	Histone H1.0 (Histone H1') (Histone H1(0)) (MyD196) [Cleaved into: Histone H1.0, N-terminally processed]
379	Histone H1.1 (H1 VAR.3) (Histone H1a) (H1a)
380	Histone H1.3 (H1 VAR.4) (H1d)
381	Histone H1.4 (H1 VAR.2) (H1e)
382	Histone H1.5 (H1 VAR.5) (H1b)
383	Histone H2A deubiquitinase MYSM1 (2A-DUB) (EC 3.4.19.-) (Myb-like, SWIRM and MPN domain-containing protein 1)
384	Histone H2A.Z/Histone H2A.V
385	Histone H2B type 1-F/J/L/Histone H2B type 1-M/Histone H2B type 1-B/Histone H2B type 1-H/Histone H2B type 2-B/Histone H2B type 1-C/E/G/Histone H2B type 1-K/Histone H2B type 1-P
386	Histone H2B type 2-E/Histone H2B type 3-B/Histone H2B type 3-A
387	Histone H3.2
388	Histone H3.3C/Histone H3.3
389	Histone H4
390	Histone-binding protein RBBP4/Histone-binding protein RBBP7
391	Histone-lysine N-methyltransferase NSD3 (EC 2.1.1.43) (Nuclear SET domain-containing protein 3) (Wolf-Hirschhorn syndrome candidate 1-like protein 1 homolog) (WHSC1-like protein 1)
392	Histone-lysine N-methyltransferase, H3 lysine-36 and H4 lysine-20 specific (EC 2.1.1.43) (H3-K36-HMTase) (H4-K20-HMTase) (Nuclear receptor-binding SET domain-containing protein 1) (NR-binding SET domain-containing protein)
393	Hsc70-interacting protein (Hip) (Protein FAM10A1) (Protein ST13 homolog)
394	Hydroperoxide isomerase ALOXE3 (EC 5.4.4.7) (Epidermis-type lipoxygenase 3) (Epidermal LOX-3) (e-LOX-3) (eLOX-3) (Hydroperoxy icosatetraenoate dehydratase) (EC 4.2.1.152)
395	Hypoxia up-regulated protein 1 (GRP-170) (140 kDa Ca(2+)-binding protein) (CBP-140)
396	Importin subunit beta-1 (Karyopherin subunit beta-1) (Nuclear factor p97) (Pore targeting complex 97 kDa subunit) (PTAC97) (SCG)
397	Inositol 1,4,5-trisphosphate receptor type 1 (IP3 receptor isoform 1) (IP3R 1) (InsP3R1) (Inositol 1,4,5-trisphosphate-binding protein P400) (Protein PCD-6) (Purkinje cell protein 1) (Type 1 inositol 1,4,5-trisphosphate receptor) (Type 1 InsP3 receptor)
398	Inositol monophosphatase 3 (IMP 3) (IMPase 3) (EC 3.1.3.25) (EC 3.1.3.7) (Golgi 3-prime phosphoadenosine 5-prime phosphate 3-prime phosphatase) (Golgi-resident PAP phosphatase) (gPAPP) (Inositol monophosphatase domain-containing protein 1) (Inositol-1(or 4)-monophosphatase 3) (Myo-inositol monophosphatase A3)
399	Insulin receptor substrate 4 (IRS-4) (Phosphoprotein of 160 kDa) (pp160)
400	Insulin-degrading enzyme (EC 3.4.24.56) (Insulin protease) (Insulinase) (Insulysin)

401	Insulin-like growth factor-binding protein 2 (IBP-2) (IGF-binding protein 2) (IGFBP-2) (mIGFBP-2)
402	Insulin-like growth factor-binding protein 5 (IBP-5) (IGF-binding protein 5) (IGFBP-5)
403	Integrator complex subunit 3 (Int3) (SOSS complex subunit A) (Sensor of single-strand DNA complex subunit A) (SOSS-A) (Sensor of ssDNA subunit A)
404	Integrin alpha-6 (CD49 antigen-like family member F) (VLA-6) (CD antigen CD49f) [Cleaved into: Integrin alpha-6 heavy chain; Integrin alpha-6 light chain]
405	Integrin alpha-M (CD11 antigen-like family member B) (CR-3 alpha chain) (Cell surface glycoprotein MAC-1 subunit alpha) (Leukocyte adhesion receptor MO1) (CD antigen CD11b)
406	Integrin beta-1 (Fibronectin receptor subunit beta) (VLA-4 subunit beta) (CD antigen CD29)
407	Interleukin enhancer-binding factor 2 (Nuclear factor of activated T-cells 45 kDa)
408	Interleukin enhancer-binding factor 3
409	Interphotoreceptor matrix proteoglycan 2 (Sialoprotein associated with cones and rods proteoglycan) (Spacrcan)
410	Intersectin-2 (EH domain and SH3 domain regulator of endocytosis 2) (EH and SH3 domains protein 2) (SH3 domain-containing protein 1B)
411	Isocitrate dehydrogenase [NAD] subunit alpha, mitochondrial (EC 1.1.1.41) (Isocitric dehydrogenase subunit alpha) (NAD(+)-specific ICDH subunit alpha)
412	Isocitrate dehydrogenase [NADP] cytoplasmic (IDH) (EC 1.1.1.42) (Cytosolic NADP-isocitrate dehydrogenase) (IDP) (NADP(+)-specific ICDH) (Oxalosuccinate decarboxylase)
413	Isocitrate dehydrogenase [NADP], mitochondrial (IDH) (EC 1.1.1.42) (ICD-M) (IDP) (NADP(+)-specific ICDH) (Oxalosuccinate decarboxylase)
414	Kelch-like protein 29 (Kelch repeat and BTB domain-containing protein 9)
415	Keratin, type I cytoskeletal 10 (56 kDa cytokeratin) (Cytokeratin-10) (CK-10) (Keratin, type I cytoskeletal 59 kDa) (Keratin-10) (K10)
416	Keratin, type I cytoskeletal 24 (Cytokeratin-24) (CK-24) (Keratin-24) (K24) (Type I keratin-24)
417	Keratin, type II cytoskeletal 1 (67 kDa cytokeratin) (Cytokeratin-1) (CK-1) (Keratin-1) (K1) (Type-II keratin Kb1)
418	Keratin, type II cytoskeletal 1b (Cytokeratin-1B) (CK-1B) (Embryonic type II keratin-1) (Keratin-77) (K77) (Type-II keratin Kb39)
419	Keratin, type II cytoskeletal 2 epidermal (Cytokeratin-2e) (CK-2e) (Epithelial keratin-2e) (Keratin-2 epidermis) (Keratin-2e) (K2e) (Type-II keratin Kb2)
420	Keratin, type II cytoskeletal 2 oral (Keratin-76) (K76) (Type-II keratin Kb9)
421	Keratin, type II cytoskeletal 5 (Cytokeratin-5) (CK-5) (Keratin-5) (K5) (Type-II keratin Kb5)
422	Keratin, type II cytoskeletal 73 (Cytokeratin-73) (CK-73) (Keratin-73) (K73) (Type II inner root sheath-specific keratin-K6irs3) (Type-II keratin Kb36)
423	KH domain-containing, RNA-binding, signal transduction-associated protein 1 (GAP-associated tyrosine phosphoprotein p62) (Src-associated in mitosis 68 kDa protein) (Sam68) (p21 Ras GTPase-activating protein-associated p62) (p68)
424	Kinesin heavy chain isoform 5A (Kinesin heavy chain neuron-specific 1) (Neuronal kinesin heavy chain) (NKHC)
425	Kinesin heavy chain isoform 5C (Kinesin heavy chain neuron-specific 2)
426	Kinesin light chain 1 (KLC 1)

427	Kinetochore-associated protein DSN1 homolog
428	Lactadherin (MFGM) (Milk fat globule-EGF factor 8) (MFG-E8) (SED1) (Sperm surface protein SP47) (MP47)
429	Lamin-B1
430	Laminin subunit alpha-4 (Laminin-14 subunit alpha) (Laminin-8 subunit alpha) (Laminin-9 subunit alpha)
431	Lanosterol synthase (EC 5.4.99.7) (2,3-epoxysqualene--lanosterol cyclase) (Oxidosqualene--lanosterol cyclase) (OSC)
432	Leucine-rich repeat-containing protein 45
433	Leucine-rich repeat-containing protein 59
434	Liprin-beta-1 (Protein tyrosine phosphatase receptor type f polypeptide-interacting protein-binding protein 1) (PTPRF-interacting protein-binding protein 1)
435	L-lactate dehydrogenase A chain (LDH-A) (EC 1.1.1.27) (LDH muscle subunit) (LDH-M)
436	L-lactate dehydrogenase B chain (LDH-B) (EC 1.1.1.27) (LDH heart subunit) (LDH-H)
437	LRP2-binding protein
438	Lupus La protein homolog (La autoantigen homolog) (La ribonucleoprotein)
439	Lysosome membrane protein 2 (85 kDa lysosomal membrane sialoglycoprotein) (LGP85) (Lysosome membrane protein II) (LIMP II) (Scavenger receptor class B member 2)
440	Lysosome-associated membrane glycoprotein 1 (LAMP-1) (Lysosome-associated membrane protein 1) (120 kDa lysosomal membrane glycoprotein) (CD107 antigen-like family member A) (LGP-120) (Lysosomal membrane glycoprotein A) (LGP-A) (P2B) (CD antigen CD107a)
441	Lysosome-associated membrane glycoprotein 2 (LAMP-2) (Lysosome-associated membrane protein 2) (CD107 antigen-like family member B) (Lysosomal membrane glycoprotein type B) (LGP-B) (CD antigen CD107b)
442	Macrophage migration inhibitory factor (MIF) (EC 5.3.2.1) (Delayed early response protein 6) (DER6) (Glycosylation-inhibiting factor) (GIF) (L-dopachrome isomerase) (L-dopachrome tautomerase) (EC 5.3.3.12) (Phenylpyruvate tautomerase)
443	Major prion protein (PrP) (PrP27-30) (PrP33-35C) (CD antigen CD230)
444	Malate dehydrogenase, cytoplasmic (EC 1.1.1.37) (Cytosolic malate dehydrogenase)
445	Malate dehydrogenase, mitochondrial (EC 1.1.1.37)
446	Mannose-P-dolichol utilization defect 1 protein (Suppressor of Lec15 and Lec35 glycosylation mutation homolog) (SL15)
447	MARCKS-related protein (Brain protein F52) (MARCKS-like protein 1) (Macrophage myristoylated alanine-rich C kinase substrate) (Mac-MARCKS) (MacMARCKS)
448	Matrin-3
449	Meiosis-specific nuclear structural protein 1
450	Membrane-spanning 4-domains subfamily A member 6D (CD20 antigen-like 8)
451	Metabotropic glutamate receptor 5 (mGluR5)
452	Methyl-CpG-binding domain protein 3 (Methyl-CpG-binding protein MBD3)
453	Methylcytosine dioxygenase TET1 (EC 1.14.11.n2) (CXXC-type zinc finger protein 6) (Ten-eleven translocation 1 gene protein homolog)
454	MICOS complex subunit Mic60 (Mitochondrial inner membrane protein) (Mitofilin)

455	Microtubule cross-linking factor 1 (Coiled-coil domain-containing protein 165) (PAR-1-interacting protein) (SOGA family member 2)
456	Microtubule-associated protein 1B (MAP-1B) (MAP1(X)) (MAP1.2) [Cleaved into: MAP1B heavy chain; MAP1 light chain LC1]
457	Microtubule-associated protein 2 (MAP-2)
458	Microtubule-associated protein RP/EB family member 3/Microtubule-associated protein RP/EB family member 2
459	Mitochondrial chaperone BCS1 (BCS1-like protein)
460	Mitofusin-2 (EC 3.6.5.-) (Hypertension-related protein 1) (Mitochondrial assembly regulatory factor) (HSG protein) (Transmembrane GTPase MFN2)
461	Mitogen-activated protein kinase 1/Mitogen-activated protein kinase 3
462	Mitotic checkpoint serine/threonine-protein kinase BUB1 (mBUB1) (EC 2.7.11.1) (BUB1A)
463	Mitotic spindle assembly checkpoint protein MAD1 (Mitotic arrest deficient 1-like protein 1) (MAD1-like protein 1)
464	Monofunctional C1-tetrahydrofolate synthase, mitochondrial (EC 6.3.4.3) (Formyltetrahydrofolate synthetase)
465	Mth938 domain-containing protein (LI2)
466	Multifunctional protein ADE2 [Includes: Phosphoribosylaminoimidazole-succinocarboxamide synthase (EC 6.3.2.6) (SAICAR synthetase); Phosphoribosylaminoimidazole carboxylase (EC 4.1.1.21) (AIR carboxylase) (AIRC)]
467	MutS protein homolog 5
468	Myb/SANT-like DNA-binding domain-containing protein 3
469	Myelin basic protein (MBP) (Myelin A1 protein)
470	Myelin expression factor 2 (MEF-2) (MyEF-2)
471	Myelin proteolipid protein (PLP) (Lipophilin)
472	Myelin-associated glycoprotein (Siglec-4a)
473	Myoferlin (Fer-1-like protein 3)
474	Myosin light chain kinase 2, skeletal/cardiac muscle (MLCK2) (EC 2.7.11.18)
475	Myosin-10 (Cellular myosin heavy chain, type B) (Myosin heavy chain 10) (Myosin heavy chain, non-muscle IIb) (Non-muscle myosin heavy chain B) (NMMHC-B) (Non-muscle myosin heavy chain IIb) (NMMHC II-b) (NMMHC-IIB)
476	Myosin-9 (Cellular myosin heavy chain, type A) (Myosin heavy chain 9) (Myosin heavy chain, non-muscle IIa) (Non-muscle myosin heavy chain A) (NMMHC-A) (Non-muscle myosin heavy chain IIa) (NMMHC II-a) (NMMHC-IIA)
477	Myristoylated alanine-rich C-kinase substrate (MARCKS)
478	N(G),N(G)-dimethylarginine dimethylaminohydrolase 1 (DDAH-1) (Dimethylarginine dimethylaminohydrolase 1) (EC 3.5.3.18) (DDAHI) (Dimethylargininase-1)
479	Na(+)/H(+) exchange regulatory cofactor NHE-RF1 (NHERF-1) (Ezrin-radixin-moesin-binding phosphoprotein 50) (EBP50) (Regulatory cofactor of Na(+)/H(+) exchanger) (Sodium-hydrogen exchanger regulatory factor 1) (Solute carrier family 9 isoform A3 regulatory factor 1)
480	NACHT, LRR and PYD domains-containing protein 9B (NALP-delta)
481	NAD-dependent protein deacetylase sirtuin-2 (EC 3.5.1.-) (Regulatory protein SIR2 homolog 2) (SIR2-like protein 2) (mSIR2L2)

482	NADH dehydrogenase [ubiquinone] iron-sulfur protein 3, mitochondrial (EC 1.6.5.3) (EC 1.6.99.3) (Complex I-30kD) (CI-30kD) (NADH-ubiquinone oxidoreductase 30 kDa subunit)
483	NADH dehydrogenase [ubiquinone] iron-sulfur protein 5 (Complex I-15 kDa) (CI-15 kDa) (NADH-ubiquinone oxidoreductase 15 kDa subunit) [Cleaved into: NADH dehydrogenase [ubiquinone] iron-sulfur protein 5, N-terminally processed]
484	NADH-cytochrome b5 reductase 3 (B5R) (Cytochrome b5 reductase) (EC 1.6.2.2) (Diaphorase-1) [Cleaved into: NADH-cytochrome b5 reductase 3 membrane-bound form; NADH-cytochrome b5 reductase 3 soluble form]
485	NADH-cytochrome b5 reductase-like (EC 1.6.2.2)
486	NADH-ubiquinone oxidoreductase 75 kDa subunit, mitochondrial (EC 1.6.5.3) (EC 1.6.99.3) (Complex I-75kD) (CI-75kD)
487	NEDD8-conjugating enzyme Ubc12 (EC 6.3.2.-) (NEDD8 carrier protein) (NEDD8 protein ligase) (Ubiquitin-conjugating enzyme E2 M)
488	Nesprin-1 (Enaptin) (KASH domain-containing protein 1) (KASH1) (Myocyte nuclear envelope protein 1) (Myne-1) (Nuclear envelope spectrin repeat protein 1) (Synaptic nuclear envelope protein 1) (Syne-1)
489	Neural cell adhesion molecule 1 (N-CAM-1) (NCAM-1) (CD antigen CD56)
490	Neuron navigator 3 (Pore membrane and/or filament-interacting-like protein 1)
491	Neuronal membrane glycoprotein M6-a (M6a)
492	Neuronal membrane glycoprotein M6-b (M6b)
493	Neuronal proto-oncogene tyrosine-protein kinase Src/Proto-oncogene tyrosine-protein kinase LCK/Tyrosine-protein kinase HCK/Tyrosine-protein kinase Fyn
494	Neuropeptide Y receptor type 6 (NPY6-R) (Pancreatic polypeptide receptor 2) (PP2)
495	Neutral alpha-glucosidase AB (EC 3.2.1.84) (Alpha-glucosidase 2) (Glucosidase II subunit alpha)
496	NHP2-like protein 1 (Fertilization antigen 1) (FA-1) (High mobility group-like nuclear protein 2 homolog 1) (Sperm-specific antigen 1) (U4/U6.U5 small nuclear ribonucleoprotein SNU13) (U4/U6.U5 tri-snRNP 15.5 kDa protein) [Cleaved into: NHP2-like protein 1, N-terminally processed]
497	Niemann-Pick C1 protein
498	Ninein
499	N-myc-interactor (Nmi) (N-myc and STAT interactor)
500	Non-POU domain-containing octamer-binding protein (NonO protein)
501	Non-specific lipid-transfer protein (NSL-TP) (EC 2.3.1.176) (Propanoyl-CoA C-acyltransferase) (SCP-chi) (SCPX) (Sterol carrier protein 2) (SCP-2) (Sterol carrier protein X) (SCP-X)
502	N-terminal kinase-like protein (105 kDa kinase-like protein) (Mitosis-associated kinase-like protein NTKL) (SCY1-like protein 1)
503	Nuclear cap-binding protein subunit 2 (20 kDa nuclear cap-binding protein) (NCBP 20 kDa subunit) (CBP20)
504	Nuclear export mediator factor Nemf (Serologically defined colon cancer antigen 1 homolog)
505	Nuclear receptor coactivator 7
506	Nucleolar protein 4
507	Nucleolar protein 56 (Nucleolar protein 5A)
508	Nucleolin (Protein C23)

509	Nucleophosmin (NPM) (Nucleolar phosphoprotein B23) (Nucleolar protein NO38) (Numatrin)
510	Nucleoporin NUP188 homolog
511	Nucleoside diphosphate kinase A (NDK A) (NDP kinase A) (EC 2.7.4.6) (Metastasis inhibition factor NM23) (NDPK-A) (Tumor metastatic process-associated protein) (nm23-M1)
512	Nucleosome assembly protein 1-like 1 (Brain protein DN38) (NAP-1-related protein)
513	Nucleosome assembly protein 1-like 4
514	Olfactory receptor 143 (Odorant receptor K18) (Olfactory receptor 170-6) (Olfactory receptor 7A)
515	Oligosaccharyltransferase complex subunit OSTC
516	Outer dense fiber protein 2 (84 kDa outer dense fiber protein) (Cenexin) (Outer dense fiber of sperm tails protein 2)
517	Pancreas transcription factor 1 subunit alpha (Pancreas-specific transcription factor 1a) (bHLH transcription factor p48) (p48 DNA-binding subunit of transcription factor PTF1) (PTF1-p48)
518	PDZ and LIM domain protein 1 (C-terminal LIM domain protein 1) (Elfin) (LIM domain protein CLP-36)
519	Peptidyl-prolyl cis-trans isomerase A (PPIase A) (EC 5.2.1.8) (Cyclophilin A) (Cyclosporin A-binding protein) (Rotamase A) (SP18) [Cleaved into: Peptidyl-prolyl cis-trans isomerase A, N-terminally processed]
520	Peptidyl-prolyl cis-trans isomerase B (PPIase B) (EC 5.2.1.8) (CYP-S1) (Cyclophilin B) (Rotamase B) (S-cyclophilin) (SCYLP)
521	Peroxiredoxin-1 (EC 1.11.1.15) (Macrophage 23 kDa stress protein) (Osteoblast-specific factor 3) (OSF-3) (Thioredoxin peroxidase 2) (Thioredoxin-dependent peroxide reductase 2)
522	Peroxiredoxin-2 (EC 1.11.1.15) (Thiol-specific antioxidant protein) (TSA) (Thioredoxin peroxidase 1) (Thioredoxin-dependent peroxide reductase 1)
523	Peroxiredoxin-4 (EC 1.11.1.15) (Antioxidant enzyme AOE372) (Peroxiredoxin IV) (Prx-IV) (Thioredoxin peroxidase AO372) (Thioredoxin-dependent peroxide reductase A0372)
524	Peroxiredoxin-6 (EC 1.11.1.15) (1-Cys peroxiredoxin) (1-Cys PRX) (Acidic calcium-independent phospholipase A2) (aiPLA2) (EC 3.1.1.-) (Antioxidant protein 2) (Non-selenium glutathione peroxidase) (NSGPx) (EC 1.11.1.9)
525	Peroxisomal acyl-coenzyme A oxidase 1 (AOX) (EC 1.3.3.6) (Palmitoyl-CoA oxidase)
526	Peroxisomal multifunctional enzyme type 2 (MFE-2) (17-beta-hydroxysteroid dehydrogenase 4) (17-beta-HSD 4) (D-bifunctional protein) (DBP) (Multifunctional protein 2) (MPF-2) [Cleaved into: (3R)-hydroxyacyl-CoA dehydrogenase (EC 1.1.1.n12); Enoyl-CoA hydratase 2 (EC 4.2.1.107) (EC 4.2.1.119) (3-alpha,7-alpha,12-alpha-trihydroxy-5-beta-cholest-24-enoyl-CoA hydratase)]
527	PH domain leucine-rich repeat-containing protein phosphatase 2 (EC 3.1.3.16) (PH domain leucine-rich repeat-containing protein phosphatase-like) (PHLPP-like)
528	PHD finger protein 20 (Hepatocellular carcinoma-associated antigen 58 homolog)
529	Phosphate carrier protein, mitochondrial (Phosphate transport protein) (PTP) (Solute carrier family 25 member 3)
530	Phosphatidylethanolamine-binding protein 1 (PEBP-1) (HCNPpp) [Cleaved into: Hippocampal cholinergic neurostimulating peptide (HCNP)]
531	Phosphoglycerate kinase 1 (EC 2.7.2.3)

532	Phosphoglycerate mutase 1 (EC 5.4.2.11) (EC 5.4.2.4) (BPG-dependent PGAM 1) (Phosphoglycerate mutase isozyme B) (PGAM-B)
533	Phospholipid transfer protein C2CD2L (C2 domain-containing protein 2-like) (Transmembrane protein 24)
534	Phospholipid-transporting ATPase FetA (EC 3.6.3.1) (ATPase class I type 8B member 2-like protein) (ATPase class I type 8B member 5) (Flippase expressed in testis A)
535	Phospholipid-transporting ATPase IA (EC 3.6.3.1) (ATPase class I type 8A member 1) (Chromaffin granule ATPase II) (P4-ATPase flippase complex alpha subunit ATP8A1)
536	Phospholipid-transporting ATPase ID (EC 3.6.3.1) (ATPase class I type 8B member 2) (P4-ATPase flippase complex alpha subunit ATP8B2)
537	Phosphoserine aminotransferase (PSAT) (EC 2.6.1.52) (Endometrial progesterone-induced protein) (EPIP) (Phosphohydroxythreonine aminotransferase)
538	Phytanoyl-CoA hydroxylase-interacting protein-like
539	Pituitary adenylate cyclase-activating polypeptide type I receptor (PACAP type I receptor) (PACAP-R-1) (PACAP-R1)
540	Plasminogen activator inhibitor 1 RNA-binding protein (PAI1 RNA-binding protein 1) (PAI-RBP1) (SERPINE1 mRNA-binding protein 1)
541	Platelet-activating factor acetylhydrolase (PAF acetylhydrolase) (EC 3.1.1.47) (1-alkyl-2-acetylglycerophosphocholine esterase) (2-acetyl-1-alkylglycerophosphocholine esterase) (LDL-associated phospholipase A2) (LDL-PLA(2)) (PAF 2-acylhydrolase)
542	Pleiotrophin (PTN) (Heparin-binding brain mitogen) (HBBM) (Heparin-binding growth factor 8) (HBGF-8) (Heparin-binding growth-associated molecule) (HB-GAM) (Heparin-binding neutrophic factor) (HBNF) (Osteoblast-specific factor 1) (OSF-1)
543	Plexin-B2
544	PML-RARA-regulated adapter molecule 1 (PRAM-1)
545	PNMA-like protein 1
546	Poly(rC)-binding protein 1 (Alpha-CP1) (Heterogeneous nuclear ribonucleoprotein E1) (hnRNP E1)
547	Poly(rC)-binding protein 2 (Alpha-CP2) (CTBP) (CBP) (Putative heterogeneous nuclear ribonucleoprotein X) (hnRNP X)
548	Poly(rC)-binding protein 4 (Alpha-CP4)
549	Polyadenylate-binding protein 1 (PABP-1) (Poly(A)-binding protein 1)
550	Polypeptide N-acetylgalactosaminyltransferase 18 (EC 2.4.1.41) (Polypeptide GalNAc transferase 18) (GalNAc-T18) (Polypeptide GalNAc transferase-like protein 4) (GalNAc-T-like protein 4) (pp-GaNTase-like protein 4) (Polypeptide N-acetylgalactosaminyltransferase-like protein 4) (Protein-UDP acetylgalactosaminyltransferase-like protein 4) (UDP-GalNAc:polypeptide N-acetylgalactosaminyltransferase-like protein 4)
551	Polyubiquitin-B/Polyubiquitin-C/Ubiquitin-40S ribosomal protein S27a/Ubiquitin-60S ribosomal protein L40
552	Potassium voltage-gated channel subfamily D member 3 (Voltage-gated potassium channel subunit Kv4.3)
553	POU domain, class 6, transcription factor 1 (Octamer-binding transcription factor EMB) (Transcription regulatory protein MCP-1)

554	Probable ATP-dependent RNA helicase DDX17 (EC 3.6.4.13) (DEAD box protein 17)
555	Probable ATP-dependent RNA helicase DDX5 (EC 3.6.4.13) (DEAD box RNA helicase DEAD1) (mDEAD1) (DEAD box protein 5) (RNA helicase p68)
556	Probable ATP-dependent RNA helicase DDX59 (EC 3.6.4.13) (DEAD box protein 59)
557	Probable G-protein coupled receptor 33
558	Probable G-protein coupled receptor 63 (PSP24-2) (PSP24-beta)
559	Profilin-1 (Profilin I)
560	Profilin-2 (Profilin II)
561	Programmed cell death 6-interacting protein (ALG-2-interacting protein 1) (ALG-2-interacting protein X) (E2F1-inducible protein) (Eig2)
562	Prohibitin (B-cell receptor-associated protein 32) (BAP 32)
563	Prohibitin-2 (B-cell receptor-associated protein BAP37) (Repressor of estrogen receptor activity)
564	Prolargin (Proline-arginine-rich end leucine-rich repeat protein)
565	Proliferation-associated protein 2G4 (IRES-specific cellular trans-acting factor 45 kDa) (ITAF45) (Mpp1) (Proliferation-associated protein 1) (Protein p38-2G4)
566	Prolyl endopeptidase FAP (EC 3.4.21.26) (Dipeptidyl peptidase FAP) (EC 3.4.14.5) (Fibroblast activation protein alpha) (FAPalpha) (Gelatine degradation protease FAP) (EC 3.4.21.-) (Integral membrane serine protease) (Post-proline cleaving enzyme) (Serine integral membrane protease) (SIMP) (Surface-expressed protease) (Seprase) [Cleaved into: Antiplasmin-cleaving enzyme FAP, soluble form (APCE) (EC 3.4.14.5) (EC 3.4.21.-) (EC 3.4.21.26)]
567	Prosaposin (Sulfated glycoprotein 1) (SGP-1) [Cleaved into: Saposin-A; Saposin-B-Val; Saposin-B; Saposin-C; Saposin-D]
568	Prostaglandin E synthase 3 (EC 5.3.99.3) (Cytosolic prostaglandin E2 synthase) (cPGES) (Hsp90 co-chaperone) (Progesterone receptor complex p23) (Sid 3177) (Telomerase-binding protein p23)
569	Prostaglandin reductase-3 (PTGR-3) (EC 1.3.1.48) (15-oxoprostaglandin 13-reductase) (Zinc-binding alcohol dehydrogenase domain-containing protein 2)
570	Proteasome subunit alpha type-4 (EC 3.4.25.1) (Macropain subunit C9) (Multicatalytic endopeptidase complex subunit C9) (Proteasome component C9) (Proteasome subunit L)
571	Proteasome subunit alpha type-5 (EC 3.4.25.1) (Macropain zeta chain) (Multicatalytic endopeptidase complex zeta chain) (Proteasome zeta chain)
572	Proteasome subunit alpha type-6 (EC 3.4.25.1) (Macropain iota chain) (Multicatalytic endopeptidase complex iota chain) (Proteasome iota chain)
573	Proteasome subunit beta type-1 (EC 3.4.25.1) (Macropain subunit C5) (Multicatalytic endopeptidase complex subunit C5) (Proteasome component C5) (Proteasome gamma chain)
574	Proteasome subunit beta type-5 (EC 3.4.25.1) (Macropain epsilon chain) (Multicatalytic endopeptidase complex epsilon chain) (Proteasome chain 6) (Proteasome epsilon chain) (Proteasome subunit X)
575	Proteasome subunit beta type-6 (EC 3.4.25.1) (Low molecular mass protein 19) (Macropain delta chain) (Multicatalytic endopeptidase complex delta chain) (Proteasome delta chain) (Proteasome subunit Y)
576	Proteasome subunit beta type-7 (EC 3.4.25.1) (Macropain chain Z) (Multicatalytic endopeptidase complex chain Z) (Proteasome subunit Z)

577	Protein ABHD8 (EC 3.-.-.-) (Alpha/beta hydrolase domain-containing protein 8) (Abhydrolase domain-containing protein 8)
578	Protein arginine N-methyltransferase 1 (EC 2.1.1.319) (Histone-arginine N-methyltransferase PRMT1)
579	Protein CIP2A (Cancerous inhibitor of PP2A) (p90 autoantigen homolog)
580	Protein DGCR14 (DiGeorge syndrome critical region 14 homolog) (ES2 protein) (Expressed sequence 2 embryonic lethal)
581	Protein disulfide-isomerase (PDI) (EC 5.3.4.1) (Cellular thyroid hormone-binding protein) (Endoplasmic reticulum resident protein 59) (ER protein 59) (ERp59) (Prolyl 4-hydroxylase subunit beta) (p55)
582	Protein disulfide-isomerase A3 (EC 5.3.4.1) (58 kDa glucose-regulated protein) (58 kDa microsomal protein) (p58) (Disulfide isomerase ER-60) (Endoplasmic reticulum resident protein 57) (ER protein 57) (ERp57) (Endoplasmic reticulum resident protein 60) (ER protein 60) (ERp60)
583	Protein disulfide-isomerase A6 (EC 5.3.4.1) (Thioredoxin domain-containing protein 7)
584	Protein DJ-1 (DJ-1) (Parkinson disease protein 7 homolog) (Protein deglycase DJ-1) (EC 3.1.2.-) (EC 3.5.1.124)
585	Protein JBTS17 (Protein C5orf42 homolog)
586	Protein kinase C delta type (EC 2.7.11.13) (Tyrosine-protein kinase PRKCD) (EC 2.7.10.2) (nPKC-delta) [Cleaved into: Protein kinase C delta type regulatory subunit; Protein kinase C delta type catalytic subunit (Sphingosine-dependent protein kinase-1) (SDK1)]
587	Protein NLRC5
588	Protein O-linked-mannose beta-1,2-N-acetylglucosaminyltransferase 1 (POMGnT1) (EC 2.4.1.-)
589	Protein RCC2
590	Protein sel-1 homolog 1 (Suppressor of lin-12-like protein 1) (Sel-1L)
591	Protein SET (Phosphatase 2A inhibitor I2PP2A) (I-2PP2A) (Template-activating factor I) (TAF-I)
592	Protein SMG9
593	Protein Spindly (Coiled-coil domain-containing protein 99) (Spindle apparatus coiled-coil domain-containing protein 1)
594	Protein transport protein Sec61 subunit alpha isoform 1 (Sec61 alpha-1)
595	Protein tweety homolog 1 (mTTY1)
596	Puromycin-sensitive aminopeptidase (PSA) (EC 3.4.11.14) (Cytosol alanyl aminopeptidase) (AAP-S)
597	Putative adenosylhomocysteinase 3/S-adenosylhomocysteine hydrolase-like protein 1
598	Putative hexokinase HKDC1 (EC 2.7.1.1) (Hexokinase domain-containing protein 1)
599	Putative protein arginine N-methyltransferase 9 (Putative protein arginine N-methyltransferase 10) (EC 2.1.1.-)
600	PWWP domain-containing protein 2A
601	Pyrroline-5-carboxylate reductase 2 (P5C reductase 2) (P5CR 2) (EC 1.5.1.2)
602	Pyruvate dehydrogenase E1 component subunit alpha, somatic form, mitochondrial (EC 1.2.4.1) (PDHE1-A type I)
603	Pyruvate dehydrogenase E1 component subunit beta, mitochondrial (PDHE1-B) (EC 1.2.4.1)
604	Pyruvate kinase PKM (EC 2.7.1.40) (Pyruvate kinase muscle isozyme)

605	Rab GDP dissociation inhibitor alpha (Rab GDI alpha) (Guanosine diphosphate dissociation inhibitor 1) (GDI-1)
606	Rab GDP dissociation inhibitor beta (Rab GDI beta) (GDI-3) (Guanosine diphosphate dissociation inhibitor 2) (GDI-2)
607	Radixin (ESP10)
608	Ran-specific GTPase-activating protein (HpaII tiny fragments locus 9a protein) (Ran-binding protein 1) (RANBP1)
609	Ras-related C3 botulinum toxin substrate 1 (p21-Rac1)
610	Ras-related protein Rab-11B/Ras-related protein Rab-11A
611	Ras-related protein Rab-14
612	Ras-related protein Rab-18
613	Ras-related protein Rab-1B
614	Ras-related protein Rab-2A
615	Ras-related protein Rab-33B
616	Ras-related protein Rab-3A
617	Ras-related protein Rab-5C
618	Ras-related protein Rab-6A (Rab-6)
619	Ras-related protein Rab-7a
620	Ras-related protein Rab-8A/Ras-related protein Rab-8B/Ras-related protein Rab-35/Ras-related protein Rab-15
621	Ras-related protein Ral-A
622	Ras-related protein Rap-1A (Ras-related protein Krev-1)
623	Ras-related protein Rap-2a
624	Receptor of activated protein C kinase 1 (12-3) (Guanine nucleotide-binding protein subunit beta-2-like 1) (Receptor for activated C kinase) (Receptor of activated protein kinase C 1) (p205) [Cleaved into: Receptor of activated protein C kinase 1, N-terminally processed (Guanine nucleotide-binding protein subunit beta-2-like 1, N-terminally processed)]
625	Receptor-type tyrosine-protein phosphatase S (R-PTP-S) (EC 3.1.3.48) (PTPNU-3) (Receptor-type tyrosine-protein phosphatase sigma) (R-PTP-sigma)
626	Regulator of G-protein signaling 4 (RGS4)
627	Replication factor C subunit 1 (A1-P145) (Activator 1 140 kDa subunit) (A1 140 kDa subunit) (Activator 1 large subunit) (Activator 1 subunit 1) (Differentiation-specific element-binding protein) (ISRE-binding protein) (Replication factor C 140 kDa subunit) (RF-C 140 kDa subunit) (RFC140) (Replication factor C large subunit)
628	REST corepressor 3
629	Reticulon-3
630	Reticulon-4 (Neurite outgrowth inhibitor) (Nogo protein)
631	Reticulon-4 receptor (Nogo receptor) (NgR) (Nogo-66 receptor) (Nogo66 receptor-1) (NgR1)
632	Retinoic acid receptor RXR-alpha (Nuclear receptor subfamily 2 group B member 1) (Retinoid X receptor alpha)
633	Retrotransposon-like protein 1 (Mammalian retrotransposon derived protein 1) (Paternally expressed gene 11 protein) (Retrotransposon-derived protein PEG11)
634	Rho GDP-dissociation inhibitor 1 (Rho GDI 1) (GDI-1) (Rho-GDI alpha)
635	Rho GTPase-activating protein 29 (Rho-type GTPase-activating protein 29)

636	Rho guanine nucleotide exchange factor 25 (Guanine nucleotide exchange factor GEFT) (Rac/Cdc42/Rho exchange factor GEFT) (RhoA/Rac/Cdc42 guanine nucleotide exchange factor GEFT) (p63RhoGEF)
637	Ribonuclease P protein subunit p21 (RNaseP protein p21) (EC 3.1.26.5) (Ribonucleoprotein V)
638	Ribonuclease P protein subunit p25 (RNase P protein subunit p25) (EC 3.1.26.5)
639	Ribosomal protein S6 kinase beta-1 (S6K-beta-1) (S6K1) (EC 2.7.11.1) (70 kDa ribosomal protein S6 kinase 1) (P70S6K1) (p70-S6K 1) (Ribosomal protein S6 kinase I) (S6K) (p70 ribosomal S6 kinase alpha) (p70 S6 kinase alpha) (p70 S6K-alpha) (p70 S6KA)
640	RING finger protein 32 (Limb region protein 2)
641	RNA binding motif protein, X-linked-like-1/RNA-binding motif protein, X chromosome
642	RNA demethylase ALKBH5 (EC 1.14.11.-) (Alkylated DNA repair protein alkB homolog 5) (Alpha-ketoglutarate-dependent dioxygenase alkB homolog 5)
643	RNA-binding protein 39 (Coactivator of activating protein 1 and estrogen receptors) (Coactivator of AP-1 and ERs) (RNA-binding motif protein 39) (RNA-binding region-containing protein 2) (Transcription coactivator CAPER)
644	RNA-binding protein 40 (RNA-binding motif protein 40) (RNA-binding region-containing protein 3)
645	RNA-binding protein FUS (Protein pigpen)
646	RNA-binding protein Raly (Maternally-expressed hnRNP C-related protein) (hnRNP associated with lethal yellow protein)
647	R-spondin-1 (Cysteine-rich and single thrombospondin domain-containing protein 3) (Cristin-3) (mCristin-3) (Roof plate-specific spondin-1)
648	RUN and FYVE domain-containing protein 1 (Rab4-interacting protein)
649	RUN and FYVE domain-containing protein 2 (Leucine zipper FYVE-finger protein) (LZ-FYVE)
650	Ryanodine receptor 1 (RYR-1) (RyR1) (Skeletal muscle calcium release channel) (Skeletal muscle ryanodine receptor) (Skeletal muscle-type ryanodine receptor) (Type 1 ryanodine receptor)
651	Sarcoplasmic/endoplasmic reticulum calcium ATPase 2 (SERCA2) (SR Ca(2+)-ATPase 2) (EC 3.6.3.8) (Calcium pump 2) (Calcium-transporting ATPase sarcoplasmic reticulum type, slow twitch skeletal muscle isoform) (Endoplasmic reticulum class 1/2 Ca(2+) ATPase)
652	Selenide, water dikinase 2 (EC 2.7.9.3) (Selenium donor protein 2) (Selenophosphate synthase 2)
653	Selenocysteine insertion sequence-binding protein 2-like (SECIS-binding protein 2-like)
654	Serine/arginine-rich splicing factor 1 (ASF/SF2) (Pre-mRNA-splicing factor SRp30a) (Splicing factor, arginine/serine-rich 1)
655	Serine/arginine-rich splicing factor 2 (Protein PR264) (Putative myelin regulatory factor 1) (MRF-1) (Splicing component, 35 kDa) (Splicing factor SC35) (SC-35) (Splicing factor, arginine/serine-rich 2)
656	Serine/arginine-rich splicing factor 3 (Pre-mRNA-splicing factor SRP20) (Protein X16) (Splicing factor, arginine/serine-rich 3)
657	Serine/arginine-rich splicing factor 4 (Splicing factor, arginine/serine-rich 4)
658	Serine/arginine-rich splicing factor 7 (Splicing factor, arginine/serine-rich 7)

659	Serine/threonine-protein kinase greatwall (GW) (GWL) (EC 2.7.11.1) (Microtubule-associated serine/threonine-protein kinase-like) (MAST-L)
660	Serine/threonine-protein kinase LATS1 (EC 2.7.11.1) (Large tumor suppressor homolog 1) (WARTS protein kinase)
661	Serine/threonine-protein kinase Nek1 (EC 2.7.11.1) (Never in mitosis A-related kinase 1) (NimA-related protein kinase 1)
662	Serine/threonine-protein kinase RIO3 (EC 2.7.11.1) (RIO kinase 3)
663	Serine/threonine-protein phosphatase 2A 65 kDa regulatory subunit A alpha isoform (PP2A subunit A isoform PR65-alpha) (PP2A subunit A isoform R1-alpha)
664	Serine/threonine-protein phosphatase 2A catalytic subunit alpha isoform (PP2A-alpha) (EC 3.1.3.16)
665	Serine/threonine-protein phosphatase 2B catalytic subunit beta isoform/Serine/threonine-protein phosphatase 2B catalytic subunit gamma isoform/Serine/threonine-protein phosphatase 2B catalytic subunit alpha isoform
666	Serine/threonine-protein phosphatase PP1-alpha catalytic subunit/Serine/threonine-protein phosphatase PP1-beta catalytic subunit/Serine/threonine-protein phosphatase PP1-gamma catalytic subunit
667	Serum albumin
668	S-formylglutathione hydrolase (FGH) (EC 3.1.2.12) (Esterase 10) (Esterase D) (Sid 478)
669	SH3 and multiple ankyrin repeat domains protein 3 (Shank3) (Proline-rich synapse-associated protein 2) (ProSAP2) (SPANK-2)
670	SH3 domain-containing kinase-binding protein 1 (Regulator of ubiquitous kinase) (Ruk) (SH3-containing, expressed in tumorigenic astrocytes)
671	Short transient receptor potential channel 4-associated protein (Trp4-associated protein) (Trpc4-associated protein) (Protein TAP1) (Rabex-5/Rin2-interacting protein) (TNF-receptor ubiquitous scaffolding/signaling protein) (Protein TRUSS)
672	Sin3 histone deacetylase corepressor complex component SDS3 (Suppressor of defective silencing 3 protein homolog)
673	SLIT and NTRK-like protein 2
674	SLIT-ROBO Rho GTPase-activating protein 1 (srGAP1) (Rho GTPase-activating protein 13)
675	Small nuclear ribonucleoprotein Sm D1 (Sm-D1) (Sm-D autoantigen) (snRNP core protein D1)
676	Sodium- and chloride-dependent glycine transporter 2 (GlyT-2) (GlyT2) (Solute carrier family 6 member 5)
677	Sodium/potassium-transporting ATPase subunit alpha-1 (Na(+)/K(+) ATPase alpha-1 subunit) (EC 3.6.3.9) (Sodium pump subunit alpha-1)
678	Sodium/potassium-transporting ATPase subunit alpha-2 (Na(+)/K(+) ATPase alpha-2 subunit) (EC 3.6.3.9) (Na(+)/K(+) ATPase alpha(+) subunit) (Sodium pump subunit alpha-2)
679	Sodium/potassium-transporting ATPase subunit beta-1-interacting protein 4 (Na(+)/K(+)-transporting ATPase subunit beta-1-interacting protein 4) (Protein FAM77A)
680	Solute carrier family 12 member 9 (Cation-chloride cotransporter-interacting protein 1) (Potassium-chloride transporter 9)
681	Solute carrier family 26 member 6 (Anion exchange transporter) (Chloride-formate exchanger) (Pendrin-L1) (Pendrin-like protein 1) (Putative anion transporter-1) (Pat-1)

682	Solute carrier organic anion transporter family member 1B2 (Liver-specific organic anion transporter 1) (LST-1) (SLC21A6) (Solute carrier family 21 member 10)
683	Solute carrier organic anion transporter family member 4C1 (Oatp-R) (Solute carrier family 21 member 20)
684	Sorbin and SH3 domain-containing protein 1 (Ponsin) (SH3 domain protein 5) (SH3P12) (c-Cbl-associated protein) (CAP)
685	Sorting nexin-4
686	Sorting nexin-7
687	SPARC (Basement-membrane protein 40) (BM-40) (Osteonectin) (ON) (Secreted protein acidic and rich in cysteine)
688	Spectrin alpha chain, non-erythrocytic 1 (Alpha-II spectrin) (Fodrin alpha chain)
689	Spectrin beta chain, non-erythrocytic 1 (Beta-II spectrin) (Embryonic liver fodrin) (Fodrin beta chain)
690	Sperm motility kinase Y (EC 2.7.11.1)
691	Sperm-associated antigen 1 (Infertility-related sperm protein Spag-1) (TPR-containing protein involved in spermatogenesis) (TPIS)
692	Spermatogenesis-associated protein 19, mitochondrial (Spermatogenic cell-specific gene 1 protein) (Spergen-1)
693	Sphingosine-1-phosphate lyase 1 (S1PL) (SP-lyase 1) (SPL 1) (mSPL) (EC 4.1.2.27) (Sphingosine-1-phosphate aldolase)
694	Spindle assembly abnormal protein 6 homolog
695	Spliceosome RNA helicase Ddx39b (EC 3.6.4.13) (56 kDa U2AF65-associated protein) (DEAD box protein UAP56) (HLA-B-associated transcript 1 protein)
696	Splicing factor U2AF 65 kDa subunit (U2 auxiliary factor 65 kDa subunit) (U2 snRNP auxiliary factor large subunit)
697	Splicing factor, proline- and glutamine-rich (DNA-binding p52/p100 complex, 100 kDa subunit) (Polypyrimidine tract-binding protein-associated-splicing factor) (PSF) (PTB-associated-splicing factor)
698	Stathmin domain-containing protein 1
699	STE20/SPS1-related proline-alanine-rich protein kinase (Ste-20-related kinase) (EC 2.7.11.1) (Serine/threonine-protein kinase 39)
700	Stress-70 protein, mitochondrial (75 kDa glucose-regulated protein) (GRP-75) (Heat shock 70 kDa protein 9) (Mortalin) (Peptide-binding protein 74) (PBP74) (p66 MOT)
701	Stress-induced-phosphoprotein 1 (STI1) (mSTI1) (Hsc70/Hsp90-organizing protein) (Hop)
702	Striatin-4 (Zinedin)
703	Structural maintenance of chromosomes protein 1A (SMC protein 1A) (SMC-1-alpha) (SMC-1A) (Chromosome segregation protein SmcB) (Sb1.8)
704	Structural maintenance of chromosomes protein 2 (SMC protein 2) (SMC-2) (Chromosome-associated protein E) (FGF-inducible protein 16) (XCAP-E homolog)
705	Structural maintenance of chromosomes protein 3 (SMC protein 3) (SMC-3) (Basement membrane-associated chondroitin proteoglycan) (Bamacan) (Chondroitin sulfate proteoglycan 6) (Chromosome segregation protein SmcD) (Mad member-interacting protein 1)
706	Succinate dehydrogenase [ubiquinone] flavoprotein subunit, mitochondrial (EC 1.3.5.1) (Flavoprotein subunit of complex II) (Fp)
707	Succinate dehydrogenase [ubiquinone] iron-sulfur subunit, mitochondrial (EC 1.3.5.1) (Iron-sulfur subunit of complex II) (Ip)

708	Succinate--CoA ligase [ADP-forming] subunit beta, mitochondrial (EC 6.2.1.5) (ATP-specific succinyl-CoA synthetase subunit beta) (A-SCS) (Succinyl-CoA synthetase beta-A chain) (SCS-betaA)
709	Succinate-semialdehyde dehydrogenase, mitochondrial (EC 1.2.1.24) (Aldehyde dehydrogenase family 5 member A1) (NAD(+)-dependent succinic semialdehyde dehydrogenase)
710	Sulfatase-modifying factor 1 (EC 1.8.99.-) (C-alpha-formylglycine-generating enzyme 1)
711	Superoxide dismutase [Cu-Zn] (EC 1.15.1.1)
712	Suppressor of cytokine signaling 7 (SOCS-7)
713	SWI/SNF-related matrix-associated actin-dependent regulator of chromatin subfamily E member 1-related (SMARCE1-related protein) (BRCA2-associated factor 35) (HMG box-containing protein 20B) (Structural DNA-binding protein BRAF35)
714	Synaptonemal complex protein 3 (SCP-3)
715	Synaptotagmin-5 (Synaptotagmin IX) (Synaptotagmin V) (SytV)
716	Syntaxin-1B
717	Syntenin-1 (Scaffold protein Pbp1) (Syndecan-binding protein 1)
718	TAR DNA-binding protein 43 (TDP-43)
719	Targeting protein for Xklp2
720	TATA box-binding protein-associated factor RNA polymerase I subunit C (RNA polymerase I-specific TBP-associated factor 95 kDa) (TAFI95) (TATA box-binding protein-associated factor 1C) (TBP-associated factor 1C) (Transcription initiation factor SL1/TIF-IB subunit C)
721	T-complex protein 1 subunit alpha (TCP-1-alpha) (CCT-alpha) (Tailless complex polypeptide 1A) (TCP-1-A) (Tailless complex polypeptide 1B) (TCP-1-B)
722	T-complex protein 1 subunit beta (TCP-1-beta) (CCT-beta)
723	T-complex protein 1 subunit delta (TCP-1-delta) (A45) (CCT-delta)
724	T-complex protein 1 subunit eta (TCP-1-eta) (CCT-eta) [Cleaved into: T-complex protein 1 subunit eta, N-terminally processed]
725	T-complex protein 1 subunit gamma (TCP-1-gamma) (CCT-gamma) (Matricin) (mTRiC-P5)
726	T-complex protein 1 subunit theta (TCP-1-theta) (CCT-theta)
727	T-complex protein 1 subunit zeta (TCP-1-zeta) (CCT-zeta-1)
728	Telomerase protein component 1 (Telomerase-associated protein 1) (Telomerase protein 1) (p240) (p80 telomerase homolog)
729	Tenascin-R (TN-R) (Janusin) (Neural recognition molecule J1-160/180) (Restrictin)
730	Thioredoxin (Trx) (ATL-derived factor) (ADF)
731	Threonylcarbamoyladenosine tRNA methylthiotransferase (EC 2.8.4.5) (CDK5 regulatory subunit-associated protein 1-like 1) (tRNA-t(6)A37 methylthiotransferase)
732	Thrombospondin-1
733	Thrombospondin-4
734	Tight junction protein ZO-2 (Tight junction protein 2) (Zona occludens protein 2) (Zonula occludens protein 2)
735	Transcription activator BRG1/Probable global transcription activator SNF2L2
736	Transcription elongation factor SPT5 (DRB sensitivity-inducing factor large subunit) (DSIF large subunit)

737	Transcription factor 7-like 2 (HMG box transcription factor 4) (T-cell-specific transcription factor 4) (T-cell factor 4) (TCF-4) (mTCF-4)
738	Transcription factor SOX-4/Protein SOX-19
739	Transcription factor SOX-5
740	Transcription intermediary factor 1-beta (TIF1-beta) (E3 SUMO-protein ligase TRIM28) (EC 2.3.2.27) (KRAB-A-interacting protein) (KRIP-1) (RING-type E3 ubiquitin transferase TIF1-beta) (Tripartite motif-containing protein 28)
741	Transcriptional activator Myb (Proto-oncogene c-Myb)
742	Transforming protein RhoA
743	Transient receptor potential cation channel subfamily A member 1 (Ankyrin-like with transmembrane domains protein 1)
744	Transitional endoplasmic reticulum ATPase (TER ATPase) (EC 3.6.4.6) (15S Mg(2+)-ATPase p97 subunit) (Valosin-containing protein) (VCP)
745	Transketolase (TK) (EC 2.2.1.1) (P68)
746	Translation initiation factor eIF-2B subunit alpha (eIF-2B GDP-GTP exchange factor subunit alpha)
747	Translationally-controlled tumor protein (TCTP) (21 kDa polypeptide) (p21) (p23)
748	Translocation protein SEC63 homolog
749	Translocon-associated protein subunit delta (TRAP-delta) (Signal sequence receptor subunit delta) (SSR-delta)
750	Transmembrane and TPR repeat-containing protein 4
751	Transmembrane emp24 domain-containing protein 10 (21 kDa transmembrane-trafficking protein) (Transmembrane protein Tmp21) (p24 family protein delta-1) (p24delta1)
752	Transmembrane emp24 domain-containing protein 9 (Glycoprotein 25L2) (p24 family protein alpha-2) (p24alpha2)
753	Transmembrane protein 33 (Protein DB83)
754	Transmembrane protein 35A (Peroxisomal membrane protein 52) (PMP52)
755	Transportin-2 (Karyopherin beta-2b)
756	Trifunctional enzyme subunit alpha, mitochondrial (TP-alpha) [Includes: Long-chain enoyl-CoA hydratase (EC 4.2.1.17); Long chain 3-hydroxyacyl-CoA dehydrogenase (EC 1.1.1.211)]
757	Trifunctional enzyme subunit beta, mitochondrial (TP-beta) [Includes: 3-ketoacyl-CoA thiolase (EC 2.3.1.16) (Acetyl-CoA acyltransferase) (Beta-ketothiolase)]
758	Triosephosphate isomerase (TIM) (EC 5.3.1.1) (Triose-phosphate isomerase)
759	Tripartite motif-containing protein 14 (PU.1-binding protein)
760	tRNA (guanine(10)-N2)-methyltransferase homolog (EC 2.1.1.-) (tRNA guanosine-2'-O-methyltransferase TRM11 homolog)
761	tRNA-splicing ligase RtcB homolog (EC 6.5.1.3) (Focal adhesion-associated protein) (FAAP)
762	Tropomodulin-2 (Neuronal tropomodulin) (N-Tmod)
763	Tropomyosin alpha-3 chain/Tropomyosin alpha-1 chain/Tropomyosin beta chain
764	Tryptophan--tRNA ligase, cytoplasmic (EC 6.1.1.2) (Tryptophanyl-tRNA synthetase) (TrpRS) [Cleaved into: T1-TrpRS; T2-TrpRS]
765	Tubulin beta-2A chain
766	Tubulin beta-2B chain
767	Tubulin beta-3 chain

768	Tubulin beta-4A chain (Tubulin beta-4 chain)
769	Tubulin beta-4B chain (Tubulin beta-2C chain)
770	Tubulin beta-5 chain
771	Tubulin beta-6 chain
772	Tubulin polyglutamylase TTLL5 (EC 6.-.-.-) (Tubulin--tyrosine ligase-like protein 5)
773	Tumor protein p73 (p53-like transcription factor) (p53-related protein)
774	Tumor susceptibility gene 101 protein (ESCRT-I complex subunit TSG101)
775	Tyrosine-protein kinase BAZ1B (EC 2.7.10.2) (Bromodomain adjacent to zinc finger domain protein 1B) (Williams syndrome transcription factor homolog) (Williams-Beuren syndrome chromosomal region 9 protein homolog)
776	Tyrosine-protein kinase Yes (EC 2.7.10.2) (Proto-oncogene c-Yes) (p61-Yes)
777	U1 small nuclear ribonucleoprotein C (U1 snRNP C) (U1-C) (U1C)
778	Ubiquitin carboxyl-terminal hydrolase 47 (EC 3.4.19.12) (Deubiquitinating enzyme 47) (Ubiquitin thioesterase 47) (Ubiquitin-specific-processing protease 47)
779	Ubiquitin carboxyl-terminal hydrolase 5 (EC 3.4.19.12) (Deubiquitinating enzyme 5) (Isopeptidase T) (Ubiquitin thioesterase 5) (Ubiquitin-specific-processing protease 5)
780	Ubiquitin carboxyl-terminal hydrolase isozyme L1 (UCH-L1) (EC 3.4.19.12) (EC 6.-.-.-) (Neuron cytoplasmic protein 9.5) (PGP 9.5) (PGP9.5) (Ubiquitin thioesterase L1)
781	Ubiquitin-conjugating enzyme E2 N (EC 2.3.2.23) (Bendless-like ubiquitin-conjugating enzyme) (E2 ubiquitin-conjugating enzyme N) (Ubc13) (Ubiquitin carrier protein N) (Ubiquitin-protein ligase N)
782	Ubiquitin-like modifier-activating enzyme 1 (EC 6.2.1.45) (Ubiquitin-activating enzyme E1) (Ubiquitin-activating enzyme E1 X) (Ubiquitin-like modifier-activating enzyme 1 X)
783	Uncharacterized protein C2orf78 homolog
784	Uncharacterized protein C5orf52 homolog
785	Unconventional myosin-X (Unconventional myosin-10)
786	Usherin (Usher syndrome type IIa protein homolog) (Usher syndrome type-2A protein homolog)
787	Vacuolar protein sorting-associated protein 41 homolog (VAM2 homolog) (mVAM2)
788	Very-long-chain 3-oxoacyl-CoA reductase (EC 1.1.1.330) (17-beta-hydroxysteroid dehydrogenase 12) (17-beta-HSD 12) (3-ketoacyl-CoA reductase) (KAR) (Estradiol 17-beta-dehydrogenase 12) (EC 1.1.1.62) (KIK-I)
789	Very-long-chain enoyl-CoA reductase (EC 1.3.1.93) (Synaptic glycoprotein SC2) (Trans-2,3-enoyl-CoA reductase) (TER)
790	Vesicle-associated membrane protein 3 (VAMP-3) (Cellubrevin) (CEB) (Synaptobrevin-3)
791	Vesicular glutamate transporter 3 (VGluT3) (Solute carrier family 17 member 8)
792	Vimentin
793	Voltage-dependent anion-selective channel protein 1 (VDAC-1) (mVDAC1) (Outer mitochondrial membrane protein porin 1) (Plasmalemmal porin) (Voltage-dependent anion-selective channel protein 5) (VDAC-5) (mVDAC5)
794	Voltage-dependent anion-selective channel protein 2 (VDAC-2) (mVDAC2) (Outer mitochondrial membrane protein porin 2) (Voltage-dependent anion-selective channel protein 6) (VDAC-6) (mVDAC6)
795	Voltage-dependent anion-selective channel protein 3 (VDAC-3) (mVDAC3) (Outer mitochondrial membrane protein porin 3)

796	Voltage-gated potassium channel subunit beta-1 (EC 1.1.1.-) (K(+) channel subunit beta-1) (Kv-beta-1)
797	V-type proton ATPase catalytic subunit A (V-ATPase subunit A) (EC 3.6.3.14) (V-ATPase 69 kDa subunit) (Vacuolar proton pump subunit alpha)
798	V-type proton ATPase subunit B, brain isoform (V-ATPase subunit B 2) (Endomembrane proton pump 58 kDa subunit) (Vacuolar proton pump subunit B 2)
799	WD repeat-containing protein 60
800	Xin actin-binding repeat-containing protein 2 (Beta-xin) (Cardiomyopathy-associated protein 3) (Myogenic MEF2-activated Xin-related protein) (Myomaxin) (mXinbeta)
801	Zinc finger and SCAN domain-containing protein 20 (Zinc finger protein 31)
802	Zinc finger E-box-binding homeobox 2 (Smad-interacting protein 1) (Zinc finger homeobox protein 1b)
803	Zinc finger MYM-type protein 4 (Zinc finger protein 262)
804	Zinc finger MYND domain-containing protein 15
805	Zinc finger protein 354A (Kidney, ischemia, and developmentally-regulated protein 1) (Renal transcription factor Kid-1) (Transcription factor 17) (TCF-17)
806	Zinc finger protein 92 (Zfp-92)
807	Zinc phosphodiesterase ELAC protein 2 (EC 3.1.26.11) (ElaC homolog protein 2) (Ribonuclease Z 2) (RNase Z 2) (tRNA 3 endonuclease 2) (tRNase Z 2)
